# Improved methods of DNA extraction from human spermatozoa that mitigate experimentally-induced oxidative DNA damage

**DOI:** 10.1371/journal.pone.0195003

**Published:** 2018-03-26

**Authors:** Miguel J. Xavier, Brett Nixon, Shaun D. Roman, Robert John Aitken

**Affiliations:** 1 Reproductive Science Group, Faculty of Science, The University of Newcastle, Callaghan, NSW, Australia; 2 Priority Research Centre for Reproductive Science, The University of Newcastle, Callaghan, NSW, Australia; 3 Priority Research Centre for Chemical Biology and Clinical Pharmacy, The University of Newcastle, Callaghan, NSW, Australia; 4 Faculty of Health and Medicine, The University of Newcastle, Callaghan, NSW, Australia; University Hospital of Münster, GERMANY

## Abstract

Current approaches for DNA extraction and fragmentation from mammalian spermatozoa provide several challenges for the investigation of the oxidative stress burden carried in the genome of male gametes. Indeed, the potential introduction of oxidative DNA damage induced by reactive oxygen species, reducing agents (dithiothreitol or beta-mercaptoethanol), and DNA shearing techniques used in the preparation of samples for chromatin immunoprecipitation and next-generation sequencing serve to cofound the reliability and accuracy of the results obtained. Here we report optimised methodology that minimises, or completely eliminates, exposure to DNA damaging compounds during extraction and fragmentation procedures. Specifically, we show that Micrococcal nuclease (MNase) digestion prior to cellular lysis generates a greater DNA yield with minimal collateral oxidation while randomly fragmenting the entire paternal genome. This modified methodology represents a significant improvement over traditional fragmentation achieved via sonication in the preparation of genomic DNA from human spermatozoa for downstream applications, such as next-generation sequencing. We also present a redesigned bioinformatic pipeline framework adjusted to correctly analyse this form of data and detect statistically relevant targets of oxidation.

## Introduction

High levels of oxidative damage in the DNA of mature humans spermatozoa is a biological feature associated with reduced fertility. This lesion is strongly correlated with paternal age and health, as well as to exposure to toxicants, reactive oxygen species (ROS) and cryostorage [1–4]. Oxidative attack generally induces the appearance of the DNA base adduct, 8-hydroxy-2’-deoxyguanosine adducts (8OHdG) [5–7]. In response to oxidative damage, spermatozoa are only capable of recruiting the first enzyme in the base excision repair (BER) pathway, 8-oxoguanine DNA glycosylase (OGG-1), which cleaves the oxidised base from the DNA to generate an abasic site that destabilises the ribose-phosphate backbone leading to a potential strand break. Since spermatozoa lack the next components of the BER pathway, apurinic endonuclease 1 (APE1) and X-ray-repair-complementing-defective-repair-in-Chinese-hamster-cells 1 (XRCC1), the abasic sites created by OGG-1 persist in the male genome. After fertilization, the oocyte, which contains these factors in abundance, continues the BER pathway in an attempt to resolve lesions in preparation for S-phase of the first mitotic division [8]. Unfortunately, the oocyte expresses OGG-1 at relatively low levels which limit its capacity to effect the removal of oxidative damage and DNA repair [9]. This situation leads to the attendant risk of increasing the mutational load carried by the offspring.

The common methodologies employed for detection of the overall DNA damage burden in spermatozoa, such as fluorophore-based assays for 8OHdG [10–12], Sperm Chromatin Structure Assay [13–15], terminal deoxynucleotidyl transferase (TdT) dUTP Nick-End Labelling (TUNEL) [16–19] and single cell gel electrophoresis (SCGE) [20–24], are capable of assessing the level of oxidative damage in individual spermatozoa but are unable of provide precise details on the specific genomic regions so affected. A small number of studies have utilised modified chromatin immunoprecipitation techniques (ChIP) [25–27] in conjunction with high-throughput sequencing [28, 29] in an attempt to identify genomic sites vulnerable to oxidative attack and subsequently correlate this information with chromatin structure and the arrangement of DNA packaging inside the nucleus of mature spermatozoa [30–34]. However, the techniques presently employed to perform DNA extraction and fragmentation from human and mouse spermatozoa make use of several compounds and procedures that increase the potential for artefactual oxidation of the DNA during sample preparation [4, 28–30, 35–37].

In order to investigate the genetic consequences of oxidative stress in human spermatozoa, it is first necessary to modify existing protocols in an effort to mitigate the introduction of confounding factors that can arise during sample preparation. In this study, we present a modified method to accurately extract oxidised DNA from human spermatozoa. In doing so, we demonstrate how, via an optimised extraction and fragmentation protocol coupled with a modified DNA immunoprecipitation, genetic samples can be prepared for next-generation sequencing and real-time quantitative PCR. Furthermore, we describe the assembly of a bioinformatic pipeline framework that use existing programs and newly constructed algorithms to identify and characterise the genomic damage inflicted by oxidative stress in human spermatozoa. The development and implementation of these modified protocols and bioinformatic framework will provide the basis to conduct future analyses into the genetic consequences of oxidative stress in sperm cells of humans and other species.

## Material and methods

### Human semen donors

Ethical approval for this study was obtained from the University of Newcastle's Human Research Ethics Committee. Samples of human semen were collected from healthy males, aged 20 to 64 years, in the University of Newcastle donor panel, with their written informed consent according to Institutional and State Government ethical approval. Collected samples were broadly classified as normozoospermic according to the World Health Organization reference values for human semen characteristics [38]. In terms of individual sample cell counts and vitality, all samples exceeded the WHO limits of 15 x 10^6^ cells/mL and 58% live cells, respectively. Donors were required to maintain a minimum of 48 h abstinence period prior to donation and to provide the sample to the laboratory staff within 1 h of ejaculation. Liquefaction of semen samples occurred at least 30 min before spermatozoa were fractionated on a discontinuous two-step Percoll gradient (80/40%) as described in Aitken et al. (2013) [39]. Motility was assessed by using phase-contrast optics at 250 x magnification and 100 cells were evaluated and any cell with a moving flagellum was classified as motile. Vitality was measured by applying the eosin exclusion test, as described previously [39]. The sperm incubation medium used in this study, except where otherwise stated, was BWW [40] supplemented with 1 mg/mL polyvinyl alcohol, 2.1 mg/mL sodium bicarbonate and 1 mg/mL glucose. Osmolarity of the media was adjusted to a range of 290–310 mOsm/kg (BWW/PVA).

### Sperm sample preparation

Human spermatozoa samples isolated via Percoll density gradient centrifugation were incubated at room temperature for 1 h with Dynabeads@ CD45 (ThermoFisher Scientific) to ensure total removal of leukocyte cells. Zymosan particles in conjunction with luminol-dependent chemiluminescence [41] were used to confirm complete removal of leukocytes. Cell counts for each individual sample were calculated prior to centrifugation and storage of samples at -80 °C as sperm pellets. Individual sperm samples collected from the same donor were pooled together, on average from three separate and consecutive donation events separated 48 h apart, to reach a minimum of 480 x10^6^ cells/mL (Fig 1.0). The pooled sample was then divided into 4 identical samples with equal volume (Fig 1.1, 1.4, 1.9 and 1.14). Divided samples were assessed again after separation to ensure that each sample contained an equal number of cells, 60 x 10^6^ cells /mL per sample, centrifuged and stored as sperm pellets at -80 °C.

**Fig 1:**
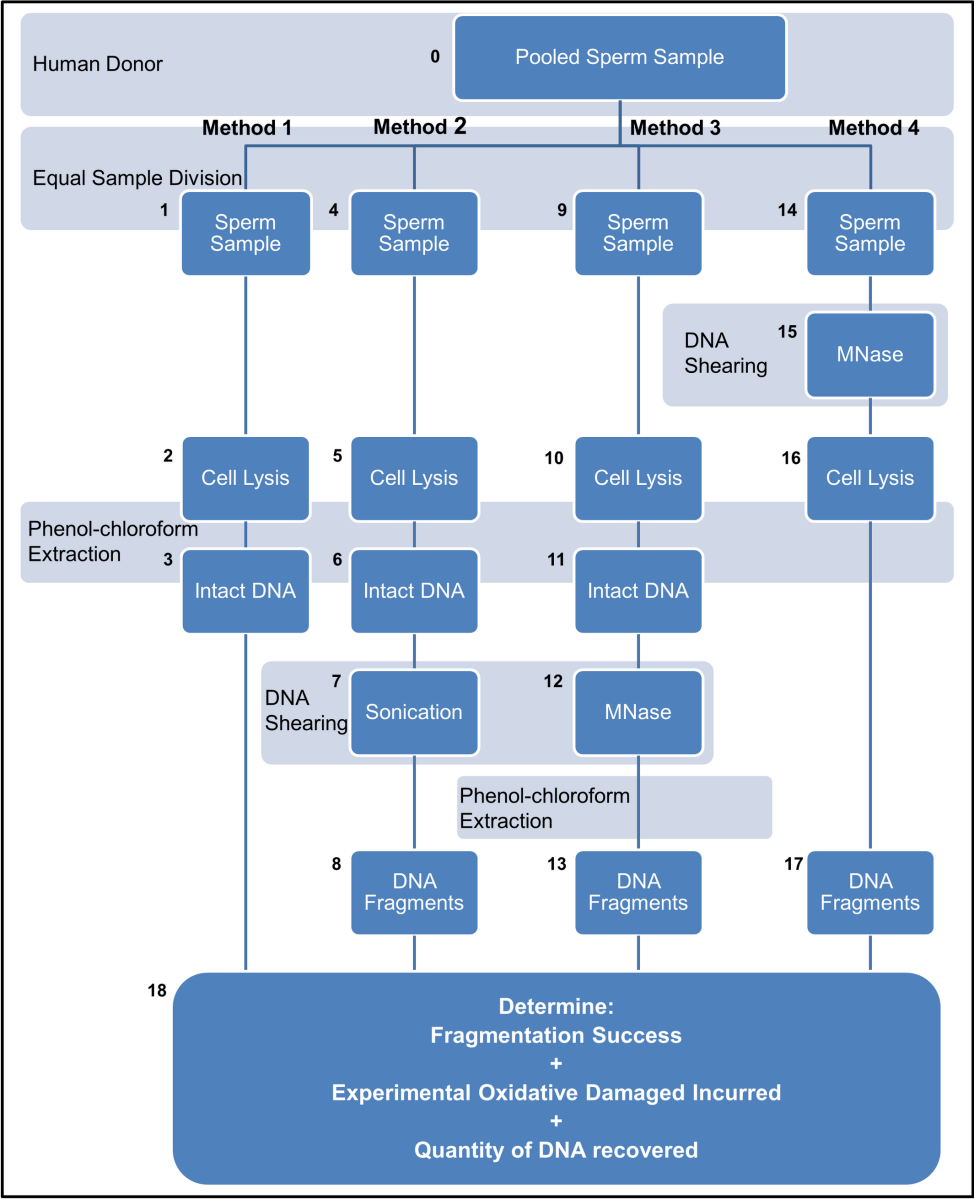
Simplified schematic detailing the modified experimental methods for DNA extraction and fragmentation from sperm cells. Human Donor spermatozoa samples, isolated via Percoll density gradient centrifugation and cleaned of leucocytes with Dynabeads, were pooled together in a single sample (0). Four identical samples with identical volume and cell numbers (1, 4, 9 and 14) were obtained from pooled sperm sample (0). DNA from sperm cells in method 1 samples (1) was collected after cell lysis (2) and DNA isolation via phenol-chloroform extraction (3) prior to comparative analysis. Samples assessed by method 2 (Sonication) (4) were subjected to cell lysis (5) followed by DNA isolation by phenol-chloroform extraction (6) and shearing by sonication (7) before comparative analysis. Samples assessed by method 3 (MNase after lysis) (9) were initially subjected to cell lysis (10) then DNA isolation by phenol-chloroform extraction (11) and DNA fragmentation by MNase digestion (12) followed by an additional DNA isolation step (13). Samples assessed by method 4 (MNase before lysis) (14) were treated with Triton X-100 and DTT prior to MNase digestion (15), sperm cells were then lysed (16) and DNA isolated via phenol-chloroform extraction (17). DNA fragment size was assessed by DNA gel electrophoresis and the amount of DNA recovered by each method was quantified with the Quant-iT PicoGreen dsDNA Kit and DNA oxidation was measured following Southern transfer and immunoblotting (18).

### Sample preparation before testing modified DNA extraction and fragmentation methods

Sperm samples were collected and prepared as described above. Pooled samples were then divided equally to generate four identical samples with 60x10^6^ cells /mL (Fig 1.1, 1.4, 1.9 and 1.14). Samples were then processed using the four methods developed for DNA extraction from spermatozoa and DNA shearing. The resulting samples of DNA (Fig 1.3, 1.8, 1.13 and 1.17) were compared to determine which method induced the least amount of collateral oxidative damage and produced the greater yield of extracted DNA. Samples in each group were processed side-by-side at the same time.

### Method 1—DNA extracted from sperm after lysis with no DNA shearing

Spermatozoa pellets of samples in the Method 1 group (Fig 1.1) were defrosted at room temperature and washed in PBS (Roche), followed by centrifugation at 400 x g for 10 min and supernatant removal. Cells were resuspended in lysis buffer (Tris-HCL 25 mM, pH 8.1, EDTA 5 mM, SDS 1%, proteinase K 0.4 mg/mL) and incubated at 55 °C for 6 h with gentle agitation. Lysates (Fig 1.2) were phenol-chloroform extracted and the DNA recovered by ethanol precipitation in the presence of ammonium acetate. DNA precipitates were resuspended in TE buffer (Tris-HCl 10mM, pH 8.1, EDTA 1mM). RNA remaining in the solution was digested with RNase A (20μg/mM) (Qiagen) at 37 °C for 20 min. Processed DNA samples were stored in TE buffer at -80 °C (Fig 1.3).

### Method 2—DNA extraction after lysis and shearing by sonication

Spermatozoa pellets of samples in the Method 2 (Sonication After Lysis) group (Fig 1.4) were defrosted at room temperature and washed in ice-cold PBS containing protease inhibitors, followed by centrifugation at 400 *g* for 10 min and supernatant removal. Cells were resuspended in lysis buffer (Tris-HCL 25 mM, pH 8.1, EDTA 5 mM, SDS 1%, proteinase K 0.4 mg/mL) and incubated at 55 °C for 6 h with gentle agitation. Lysates (Fig 1.5) were phenol-chloroform extracted and the DNA recovered by ethanol precipitation in the presence of ammonium acetate. DNA precipitates were resuspended in TE buffer (Fig 1.6). RNA present in the solution was digested with RNase A (20μg/mM) at 37 °C for 20 min. DNA in samples were sheared using sonication (Fig 1.7), samples on ice were submitted to repeated cycles of 10 s sonic pulses at high intensity with 1 min interval between pulses for six consecutive times in a Bioruptor (Diagenode). Following sonication DNA samples were centrifuged at 16,000 *g* for 10 min at 4 °C. Samples with sheared DNA were stored in TE buffer at -80 °C (Fig 1.8).

### Method 3—DNA extraction after lysis followed by MNase digestion

Spermatozoa pellets of samples in the Method 3 (MNase After Lysis) group (Fig 1.9) were defrosted at room temperature and washed in PBS, followed by centrifugation at 400 *g* for 10 min and supernatant removal. Cells were resuspended in lysis buffer (Tris-HCl 20 mM, pH 8.1, NaCl 150 mM, SDS 0.5%, proteinase K 0.4 mg/mL) and incubated at 55 °C for 6 h with gentle agitation. Lysates (Fig 1.10) were phenol-chloroform extracted and the DNA recovered by ethanol precipitation in the presence of ammonium acetate. DNA precipitates were resuspended in ice-cold PBS containing protease inhibitors and 10mM Dithiothreitol (DTT) and incubated at 37 °C for 30 min to relax disulphide bonds within and between proteins still attached to DNA (Fig 1.11). After incubation, 10 x MNase Reaction Buffer (New England Biolabs) and 100 x BSA was added to samples, before addition of 1U MNase (New England Biolabs) generated fragments between 200–2,000 bp in size (Fig 1.12). Samples were then incubated at 37 °C for 45 min with gentle agitation. DNA fragmentation was stopped by adding 5mM EGTA and incubating for a further 30 min at 37 °C. RNA present in solution was digested with RNase A (20 μg/mM) at 37 °C for 20 min. A second phenol-chloroform extraction and ethanol precipitation was conducted to recover the fragmented DNA from the solution. Pellets of fragmented DNA were resuspended in TE buffer and stored at -80 °C (Fig 1.13).

### Method 4—MNase digestion before by lysis and DNA extraction

Spermatozoa pellets of samples in the Method 4 (MNase before Lysis) group (Fig 1.14) were resuspended in 0.5% Triton X-100 in PBS containing protease inhibitors and incubated on ice for 10 min to permeabilise the cell membranes, followed by centrifugation at 400 *g* for 10 min and supernatant removal. Cells were resuspended in 0.5% Triton X-100 in PBS incubated and centrifuged as above. Spermatozoa cells were washed in ice-cold PBS, centrifuged as before with an additional 1 min at 900 *g*. Spermatozoa were then incubated at 37 °C for 30 min with ice-cold PBS containing protease inhibitors and 10 mM DTT to relax disulphide bonds within and between protamines. After incubation, 10 x MNase Reaction Buffer and 100 x BSA was added to each sample, before addition of 1U MNase. Samples were then incubated at 37 °C for 45 min with gentle agitation, to generate fragments between 200–2,000 bp in size (Fig 1.15). DNA fragmentation was stopped by adding 5 mM EGTA and incubating for a further 30 min, followed by centrifugation at 16,000 *g* for 10 min at 4 °C and discarding the supernatant. Pellets of spermatozoa with fragmented DNA were incubated at 55 °C for 6 h with gentle agitation in lysis buffer (Tris-HCl 20 mM, pH 8.1, NaCl 150 mM, SDS 0.5%, proteinase K 0.4 mg/mL). Following cell lysis, RNA present in the solution was digested with RNase A (20 μg/mM) at 37 °C for 20 min. Lysates (Fig 1.16) were phenol-chloroform extracted and the DNA recovered by ethanol precipitation in the presence of ammonium acetate. DNA precipitates were resuspended in TE buffer and stored at -80 °C (Fig 1.17).

### DNA quantification and DNA fragmentation assessment

The amount of DNA recovered from each sample processed via the four methods described above (Fig 1.3, 1.8, 1.13 and 1.17) was quantified with the Quant-iT PicoGreen dsDNA Kit (Invitrogen). DNA integrity and fragment size in samples was determined by gel electrophoresis in a 1% (w/v) agarose (Promega) gel stained with 0.4 μg/mL ethidium bromide. A 100 bp DNA ladder (Promega) was run alongside both samples as a molecular weight marker. DNA was visualised under UV illumination using a Bio-Rad Gel Doc 1000 (Bio-Rad) and photographed using a Kodak DC90 Zoom Digital Camera (Kodak) in conjunction with the Kodak 1D v3.6 image analysis software.

### Quantification of experimentally-induced oxidative DNA damage

The amount of oxidative damage inflicted to the DNA during extraction and shearing procedures employed in the methods described above (Fig 1.3, 1.8, 1.13 and 1.17) was assessed by conducting a southern transfer followed by immunoblotting. Samples subjected to electrophoresis in the agarose gels were transferred to nitrocellulose membranes with 10x saline-sodium Citrate solution (10x SSC) overnight via capillary action. Membranes were rinsed twice after transfer in 2x SSC and dried in filter paper. Transferred DNA was immobilised by exposing the dry membranes covered in filter paper to UV light for 45 min, which promoted non-covalent attachment of DNA to the membrane. Membranes were blocked in 3% BSA-TBS at room temperature for 1 h, with constant agitation. Blocked membranes were rinsed twice with dH_2_O before adding DNA/RNA Damage Antibody (Novus Biological, clone 15A3) (1:500 dilution) in antibody buffer (sodium phosphate 10 mM, pH 7, NaCl 140 mM, Triton X-100 0.05%) and were then incubated for 4 h at 4 °C, with constant rotation. Membranes were washed twice with TBS-T (Tris 100 mM. NaCl150 mM, Tween-20 0.1%) and incubated with a goat anti-mouse secondary antibody (Alexa 555, Invitrogen, 1:500 dilution) for 1 h at room temperature, with constant rotation. After incubation the membranes were washed four times in TBS-T. Immunoblots were then visualised and quantified using the commercially available ECl+Plus Chemiluminescence detection Kit (Amersham Pharmacia Biotech), the LAS-4000 Luminescence Imaging System and associated software (Fujifilm).

### Modified DNA immunoprecipitation (MoDIP) to extract oxidised DNA fragments

**Fig 2:**
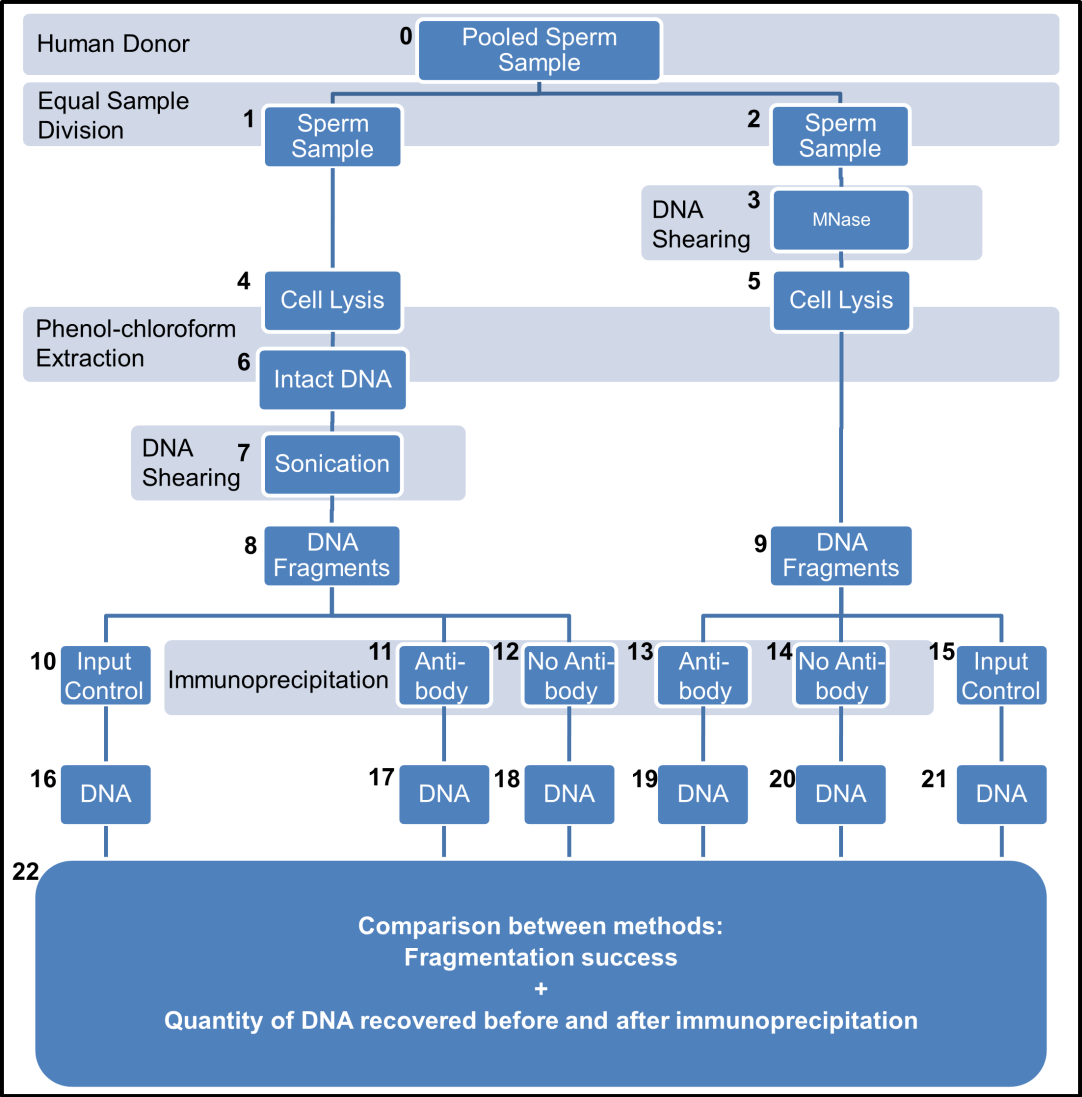
Simplified schematic detailing the modified DNA immunoprecipitation technique to isolate oxidised DNA fragments. Human Donor spermatozoa samples, isolated via Percoll density gradient centrifugation and cleaned of leucocytes with Dynabeads, were pooled together in a single sample (0). Each pooled sample was divided into two identical samples with equal volume and cell numbers (1, 2) before being processed by method 2 (sonication) (4, 6–8) or by method 4 (MNase before cell lysis) (3, 5, 9). The genomic DNA extracted and prepared following these methods was divided into 3 samples, the input control samples (10, 15), the antibody treated (11, 13) and the no-antibody negative controls (12, 14). DNA fragments in each sample was ran on a DNA gel and quantified with the Quant-iT PicoGreen dsDNA Kit (22).

Processed samples were submitted to a modified DNA immunoprecipitation (MoDIP) technique to extract oxidised DNA from the samples (Fig 2), adapted from the existing methylation DNA immunoprecipitation (MeDIP) procedure [42]. Similarly to ChIP and MeDIP techniques that use antibodies to specifically target proteins bound to DNA or methyl cytosine residues in DNA sequences, respectively, our modified procedure (MoDIP) employed the DNA/RNA Damage Antibody (Novus Biological, clone 15A3) that binds specifically to the oxidised guanine metabolite, 8-hydroxy,2’deoxyguanosine (8OHdG) to precipitate oxidised DNA fragments from the sample. To achieve this purpose, pooled sperm samples (Fig 2.0, collected and prepared as described above, were divided into two identical samples containing 60x10^6^ cells /mL (Fig 2.1 and 2.2). Corresponding sample pairs then processed using method 2 (sonication) and method 4 (MNase before cell lysis) to generated samples of fragmented DNA (Fig 2.8 and 2.9). The DNA in these samples was then denatured at 95 °C for 10 min and promptly conserved at 4 °C, at which time one tenth of the volume was taken aside to serve as the internal input control (Fig 2.10 and 2.15). The remaining sample volume was further partitioned into two equal volume portions, and diluted in ice-cold immunoprecipitation buffer (sodium phosphate 10 mM, pH 7, NaCl 140 mM, Triton X-100 0.05%) and incubated overnight at 4 °C with constant rotation in the presence of 4 μl of antibody (Fig 2.11 and 2.13) or 4 μl of dH20 (Fig 2.12 and 2.14). Protein A/G Magnetic Beads (Thermo Scientific) were added to the samples and incubated for an additional 4 h at 4 °C with constant rotation. Magnetic beads were then washed 4 times for 5 min at 4 °C in immunoprecipitation buffer. The DNA bound to the magnetic beads was eluted and purified using the commercially available IPure Kit v2 (Diagenode). DNA recovered for each sample (Fig 2.16–2.21) was ran on electrophoresis gels and quantified with the Quant-iT PicoGreen dsDNA Kit (Invitrogen).

### Bioinformatic pipeline framework to analyse sequenced data

**Fig 3:**
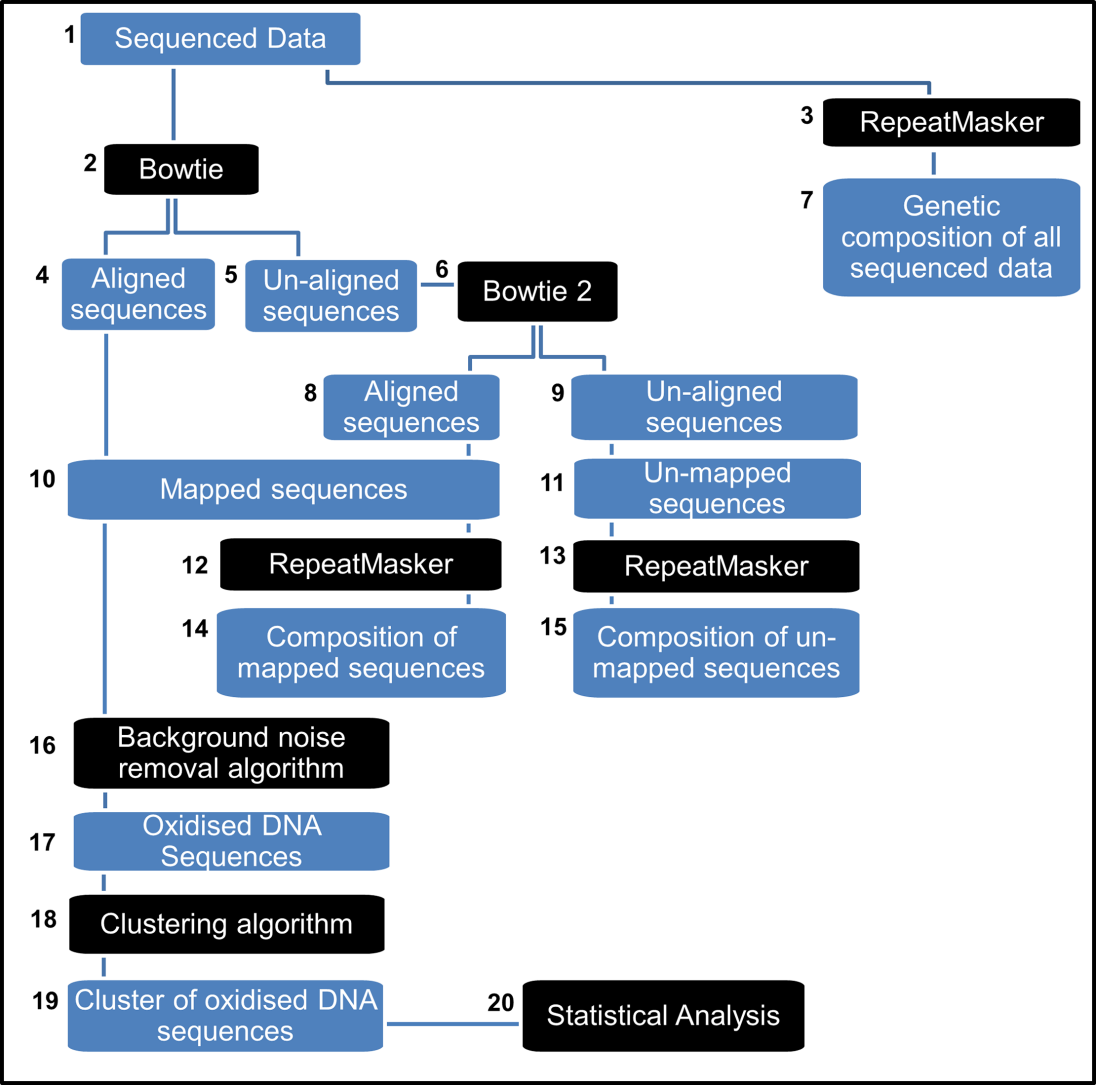
Bioinformatics pipeline framework. Mock sequencing data (1) was initially processed by Bowtie (2) to align sequences against the human reference genome. Sequences un-aligned by Bowtie (5) were submitted to Bowtie 2 (6) to resolve sequence ambiguity and increase the number of successfully mapped sequences (10). The RepeatMasker software was used to analyse sequence composition of the entire dataset (3), the mapped (12) and un-mapped sequences (13). A constructed algorithm (16) was then used to remove sequences classified as background noise in MoDIP negative controls. The remaining sequences (17) were then grouped into clusters of oxidised sequences by another newly developed algorithm (18). Cluster information (19) was imported into statistical analysis software (20) to determine the significant relevance of this data.

In preparation of obtaining sequencing results, we developed and tested a bioinformatic pipeline framework to analyse and interpret the sequencing data (Fig 3). A mock dataset was created by mining the Genome Reference Consortium Human Build 38 (GRCh38/hg39) (December 2013, NCBI) to extract one hundred DNA sequences with a length of 25 base pairs (bp) (Fig 3.1). Selected regions were sampled from chromosomes 1, 2 and X, and covered both coding and non-coding regions of DNA. Half the sequences were chosen consecutively from two 200 bp region, separated by 100 bp, so sequences overlapped for 5 bp at each end with surrounding sequences. Genomic locations for each individual sequence were kept for future reference.

Mock sequence data arranged into a single file in FASTA format was mapped against the human reference genome using Bowtie [43] (Fig 3.2). Bowtie was run twice; first in default mode and then with–n alignment and–best mode options selected, enforcing that alignments had no more than 1 mismatches in first 5 bases and to give preference to alignments with the fewer mismatches. Afterwards, a subset of 75 sequences in the mock dataset were manually modified by replacing a single nucleotide with a mismatching alternative or by deleting or inserting 5 nucleotides to the middle of the sequence, to simulate mutational changes and sequencing errors. This modified mock dataset was then processed by Bowtie 2 [44] and mapped against the human reference genome using the default parameters (Fig 3.6). The alignment results obtained from Bowtie and Bowtie 2 were further processed using a personally constructed Perl algorithm to read and extract the sequence reference name, its genomic location and DNA sequence from the output files generated, which contained additional information not relevant for our analysis (S1 File). The alignment results for each sequence were then analysed with a simple Perl script that compared the alignment positions with the known genomic positions to assess the accuracy of the mapping programs (S2 File).

Successfully mapped sequences were then sorted by chromosome and by start position by another Perl algorithm that separated aligned sequences from those with multiple alignments or too ambiguous to align to a known position. At this stage, aligned and unaligned sequence datasets were analysed with the RepeatMasker program [45] using the repeat library Repbase [46] to screen for repeat elements and characterise the genomic composition of each sequence (Fig 3.13).

Having developed the MoDIP protocol to extracted oxidised DNA fragments from samples using an antibody that specifically targets oxidised nucleotide (Fig 2.11 or 2.13), we included a set of negative control samples (Fig 2.12 or 2.14) to detect whether any DNA fragments were transferred without the use of an antibody. With negative controls expected to generate a random collection of sequences, these sequences could be classified as background inherent to the experimental protocol and need to be removed from the MoDIP results. Thus, we created another Perl algorithm (S3 File) to remove DNA sequences that were identical or that had more than 50% overlap with sequences present in the negative control samples (Fig 3.16). A small dataset of 25 sequences obtained from the human reference genome that matched or overlapped with the sequences in the unmodified mock dataset was created to simulate this step of the analysis. The algorithm was then tested to ensure that all matching and overlapping sequences were successfully removed from the dataset of mapped sequences.

At this stage of the analysis it was necessary to sort and group mapped sequences to determine the boundaries of the regions of containing oxidised DNA within them. Thus, we constructed a Perl algorithm (S4 File) to sort through the sequences and group overlapping ones to form continuous regions (Fig 3.18). The algorithm worked by first sorting the sequences by chromosome and genomic location and then by taking the start and end positions of consecutive sequences to determine whether they covered the same region of the genome. Additionally, the Perl script was designed to include an optional numerical parameter that determined the number of base pairs between two non-overlapping sequences allowed to still consider them part of the same grouping. This option was coded into the algorithm make it more dynamic and account for sequences that could either be lost by sequencing or be too ambiguous to accurately map. Two result files were produced by this script; the first file contained the genomic location of each sequence cluster assembled and detailed which sequences were inside the regions. The second file held the genomic location of the clusters and counted the number of sequences inside the cluster. Both files arranged their data into a BED format to allow visual inspection of the coverage and counts within each region in a genomic browser such as the UCSC Genome Browser [47]. The resulting cluster information could then be imported into statistical analysis software to determine which regions were significantly composed by oxidised DNA (Fig 3.20).

### Statistical analysis

DNA quantity and oxidation values from all samples were imported into R Statistical Software [48]. The amount of extracted DNA at the end of each of the multiple methods tested was used to normalise the oxidation values to allow for an accurate comparison of the collateral oxidative damage caused by the different approaches. Statistical significance of differences in DNA yield obtained from each method was determined by a one-way analysis of variance (ANOVA) followed by post-hoc Tukey’s. Statistical significance of the variance in collateral oxidative damage inflicted between methods was determined by performing a Kruskal-Wallis one-way analysis of variance and the post-hoc multiple comparison test using the kruskalmc function in the pgirmess package in R [49]. Differences in DNA extracted before and after MoDIP from Sonicated and MNase digested samples were calculated by performing Mann-Whitney U test to determine statistical significance. Cluster information obtained from the bioinformatic workflow were imported into R and analysed using the edgeR package [50, 51].

## Results

### Successful re-optimisation to reduce experimentally-induced DNA oxidation

The modified protocols developed to better investigate the impact of oxidative stress on human sperm DNA (Fig 1) were successfully adapted from existing methods for DNA extraction from spermatozoa [4, 28–30, 35–37] by completely removing or minimising the presence of oxidising chemical agents not strictly necessary for DNA extraction and fragmentation. Commonly used reducing agents were either completely removed (β–ME), or employed at lower concentrations (DTT). Initially, DTT was completely removed from all experimental approaches, however DTT and Triton X-100 were necessary to relax the highly compacted chromatin structure and permit successful MNase digestion in Method 4 (MNase before lysis). Without exposure to DTT and Triton X-100 to permeabilise and relax the chromatin structure before exposure to MNase (Fig 1.12 and 1.15), DNA fragmentation was impaired by restricting digestion to specific chromatin locations leading to the generation of a large number of DNA fragments approximately 50 kb in size, which correspond to the size of toroids; a few DNA fragments below 100bp in size and no fragments in our desired range of 200 to 2,000.

The three modified methods developed in this study (methods 2, 3, and 4) were each able to generate the DNA fragments in the range of 200–2,000 bp in size after re-optimisation. In Method 2 (sonication after lysis) DNA shearing was optimised to determine that exposure to six consecutive cycles of 10 s sonic pulses at high intensity followed by intervals of 1 min in a Bioruptor generated DNA fragments of the appropriate size range. DNA digestion by MNase used in Methods 3 and 4 (MNase after and before cell lysis) was adjusted by testing several incubation times using the recommended concentration of 1U of MNase and determining that DNA fragments in the desired range were obtained after 45 min of incubation at 37 °C with gentle agitation.

### Re-optimised methods produce identical DNA fragmentation

**Fig 4:**
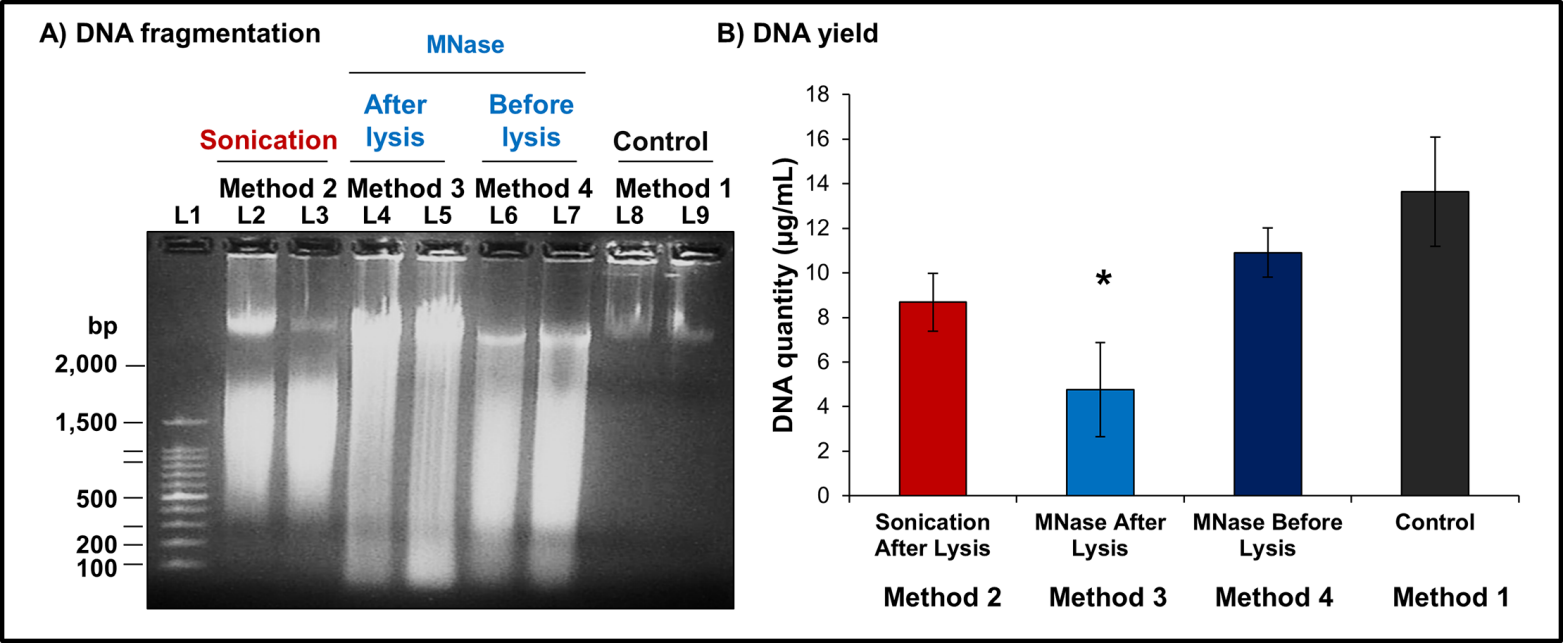
Comparative assessment of DNA fragmentation and DNA yield generated by different experimental methods. **a)** Resolution of extracted DNA via gel electrophoresis showed successful fragmentation was achieved using each experimental method. Method 2 and 4 accurately generated DNA fragments in target range of 200–2,000 bp in size. **L1:** 100bp DNA Ladder. **L2-3:** DNA fragments obtained following method 2 (sonication). **L4-5:** DNA fragments obtained following method 3 (MNase after cell lysis). **L6-7:** DNA fragments obtained following method 4 (MNase before cell lysis). **L8-9:** DNA extracted from samples using control method 1. **b)** The amount of DNA recovered from sperm cells was significantly influenced by the extraction protocol utilized (One-way ANOVA, p = 0.034, n = 6). Method 4 (MNase before cell lysis) achieved the highest DNA yield, isolating an equivalent amount of DNA to that of controls. Method 3 (MNase before cell lysis) generated a significantly lower DNA yield (Tukey’s HDS test, p = 0.02) and performed the worst among compared methods. * p<0.05.

Our three modified and re-optimised methods for DNA extraction from human spermatozoa and DNA shearing were able to consistently generate DNA fragments in the desired range of 200–2,000 bp in size (Fig 4A). In samples of the control group (method 1) intentionally not submitted to either sonication or MNase digestion, the DNA remained largely un-fragmented. The presence of large DNA fragments in control samples observed after DNA electrophoresis (Fig 4A—Lanes 8–9) were a consequence of mechanical fragmentation during the process of DNA extraction and sample loading into the DNA gel. Nonetheless the majority of the DNA in control samples remained intact and did not migrate out of the loading wells during electrophoresis.

DNA fragmentation in all three experimental methods produced equitable results in terms of fragment size independently of the technique used, both sonication and MNase digestion produced fragments between 200–2,000 bp in size (Fig 4A). Methods 2 (sonication) and 4 (MNase before cell lysis) generated similar results with the majority of fragments within the desired range (Fig 4A—Lanes 2–3, 6–7). However experimental method 3 (MNase after cell lysis) was found to be less effective in producing fragments within the 200–2,000 bp range (Fig 4A—Lanes 4–5), with fragments longer than 2,000 bp still abundantly present in these samples. Consequently, methods 2 (sonication) and 4 (MNase before lysis) were shown to outperform method 3 (MNase after lysis) in terms of DNA fragmentation success.

### Greater DNA yields achieved by methods using sonication and MNase before lysis

The amount of DNA recovered from samples submitted to the three experimental approaches was found to vary significantly (One-way ANOVA, p = 0.034, n = 6) depending on which method was used to extract and fragment the DNA (Fig 4B). Method 3 (MNase after cell lysis) produced the least amount of extracted DNA of all the methods assessed, an average of 4.8 ± 2.1 μg/mL, and achieved a significantly reduced yield of DNA (Tukey’s HDS test, p = 0.02) when compared to the amount extracted from control samples (14.6 ± 2.4 μg/mL). The quantity of DNA recovered from methods 2 (sonication) and 4 (MNase before cell lysis) was statistically similar between both approaches and controls. However, method 2 (sonication) generated a relatively lower yield compared to method 4 (MNase before lysis) with an average of 8.7 ± 1.3 and 10.9 ± 1.1 μg/mL of DNA being extracted from cells using the respective methods.

Minor differences in yield between experimental methods and controls were attributed to losses during DNA fragmentation and recovery processes, in particular in the course of multiple washing and transfer steps required to remove all cellular debris and compounds used in the fragmentation and purification of DNA. The lower yield obtained from samples using method 3 (MNase after cell lysis) (approximating one third of the amount of DNA recovered from controls) was most likely a consequence of recurrent losses during sequential phenol-chloroform extractions; one performed after cell lysis and the other following MNase digestion. Thus, method 3 (MNase after cell lysis) continued to be surpassed in terms of efficiency by both methods 2 (sonication) and 4 (MNase before cell lysis) (Fig 4B).

### MNase digestion before cell lysis significantly reduced residual oxidation

**Fig 5:**
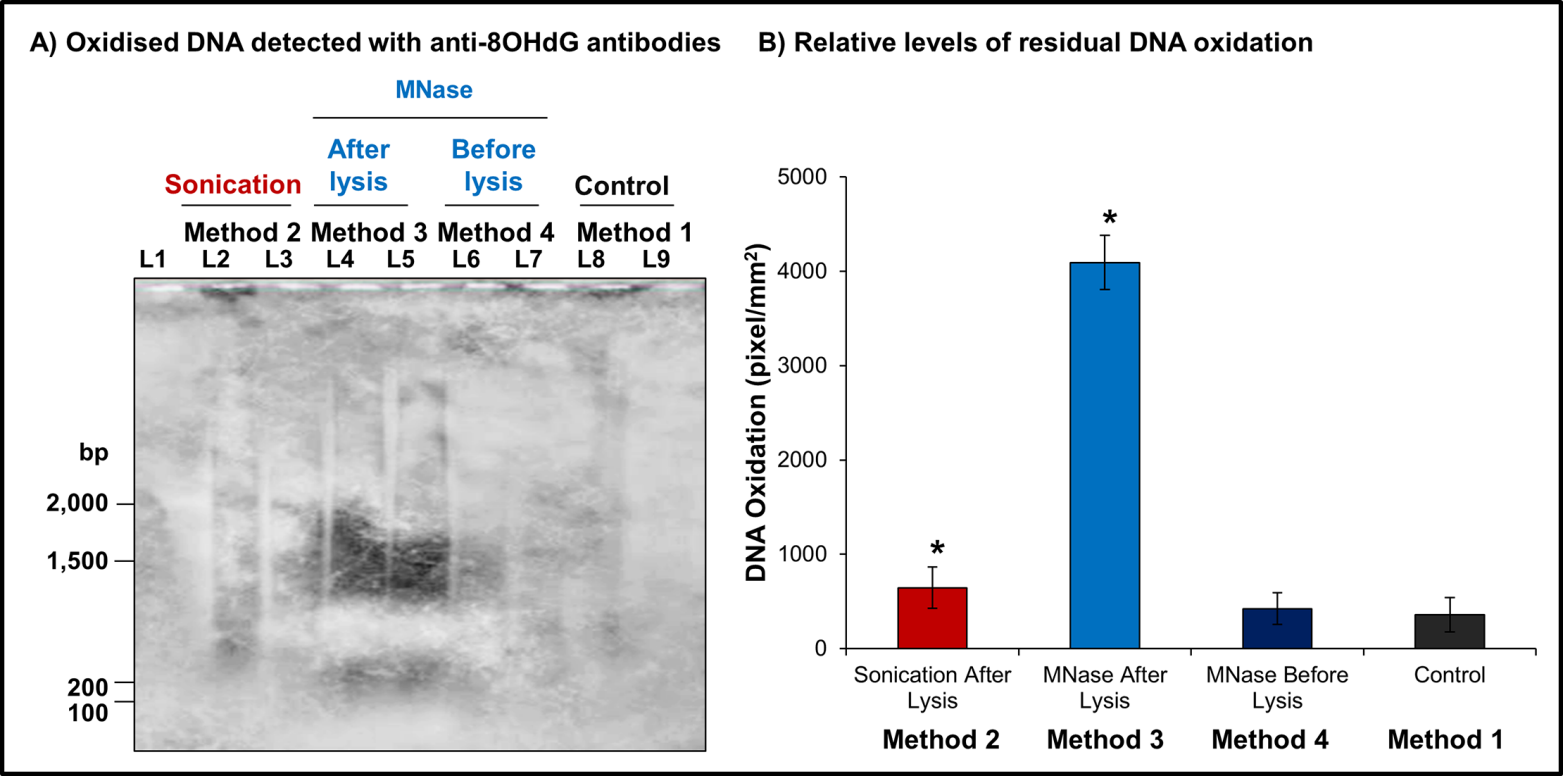
Levels of DNA oxidation induced by different methodological approaches of DNA extraction. **a)** Detection of oxidised DNA was performed following Southern transfer and sequential immunoblotting with anti-8OHdG and HRP-conjugated anti-mouse antibodies. The DNA extracted via experimental method 3 displayed the highest levels of oxidative damage. **L1:** 100bp DNA Ladder. **L2-3:** Oxidised DNA extracted via method 2 (sonication). **L4-5:** Oxidised DNA generated by method 3 (MNase after cell lysis). **L6-7:** Oxidised DNA induced by method 4 (MNase before cell lysis). **L8-9:** Oxidised DNA extracted using method 1. **b)** The amount of residual DNA oxidation was significantly affected by experimental methods used to extract and fragment sperm DNA (Kruskal-Wallis test, p = 0.04, n = 6). Multiple comparison testing revealed a significant increase in DNA oxidation (p<0.05, n = 6) in samples obtained using methods 2 (sonication) and 4 (MNase after cell lysis). *p<0.05.

The level of oxidative damage incurred by the different experimental approaches was found to vary significantly (Kruskal-Wallis test, p = 0.04, n = 6), depending on the method was used to extract and fragment the DNA (Fig 5). Using pixel intensity as a proxy for the amount of oxidatively damaged DNA, we showed that even DNA extracted from control samples (Fig 5A Lanes 8–9) less exposed to chemical and mechanical stresses contained oxidative damage (Fig 5B). Method 4 (MNase before cell lysis) demonstrated the least evidence for experimentally-induced oxidative damage to the DNA (Fig 5A Lanes 6–7) and a minimal increase compared to DNA extracted from control samples, with mean averages of 585.4 ± 118.2 and 552.7 ± 108.0 pixels/mm^2^, respectively (Fig 5B). Experimental method 1 (sonication) was found to statistically (Multiple comparisons after Kruskal-Wallis, p<0.05, n = 6) double the amount of DNA oxidation, 989.7 ± 112.2 pixels/mm^2^, as compared to control samples (Fig 5B). DNA from samples using method 3 (MNase after cell lysis) acquired the most oxidative damage during the extraction and fragmentation processes, on average 4,242.3 ± 283.6 pixels/mm^2^, most likely due to double the exposure to phenol-chloroform before and after MNase digestion. A significant increase in DNA oxidation (Multiple comparisons after Kruskal-Wallis, p<0.05, n = 6) of approximately eight times the amount of damage present in method 1 (Fig 5B), established that method 3 was not adequate to investigate the effects of oxidation on the DNA of sperm cells without adding confounding factors.

Comparatively, the best method for extracting and fragmenting DNA from human spermatozoa was method 4 (MNase before cell lysis) in which DNA incurred the least amount of damage, while achieving the best yield and proper fragment range. Method 3 (MNase after cell lysis) in contrast performed the worst with high levels of oxidation affecting the relatively lower amount of DNA extracted from cells. Although method 2 (Sonication after lysis) did not generate as much oxidatively damage DNA than method 4 (MNase after lysis), it nonetheless increased the levels of oxidation relatively to controls. Thus, employing method 4 (MNase before lysis) provided a valid alternative to existing methods while reducing any further oxidative insult.

### Modified DNA immunoprecipitation successfully extracts oxidised DNA

The findings obtained from our analyses provided us with a suggestion on which methods to use for investigating the impact of DNA oxidation in human spermatozoa. These results indicated that we had successfully designed and re-optimised two experimental protocols to minimise the loss of genetic material and the impact of experimentally-induced oxidative stress as well as other confounding factors during sample preparation. Consequently, samples of spermatozoa from healthy normozoospermic human donors (Fig 2.0) were once more divided into two identical samples containing the same number of cells (Fig 2.1 and 2.2) before extracting and fragmenting the DNA via the two methods previously identified to generate the best results, i.e. method 2 (sonication) and method 4 (MNase before cell lysis). Afterwards, the oxidised fragments of DNA were extracted from the samples by means of a modified DNA immunoprecipitation (MoDIP) strategy using an antibody to specifically target oxidised nucleotides (Fig 2.17 and 2.19). This approach also included two control samples, the input controls (Fig 2.16 and 2.21) to demonstrate that before the MoDIP procedure the samples held the entire genome composed by oxidised and un-oxidised DNA fragments; and the MoDIP negative controls (Fig 2.18 and 2.20) where no antibody was added to the samples to verify antibody specificity.

**Fig 6:**
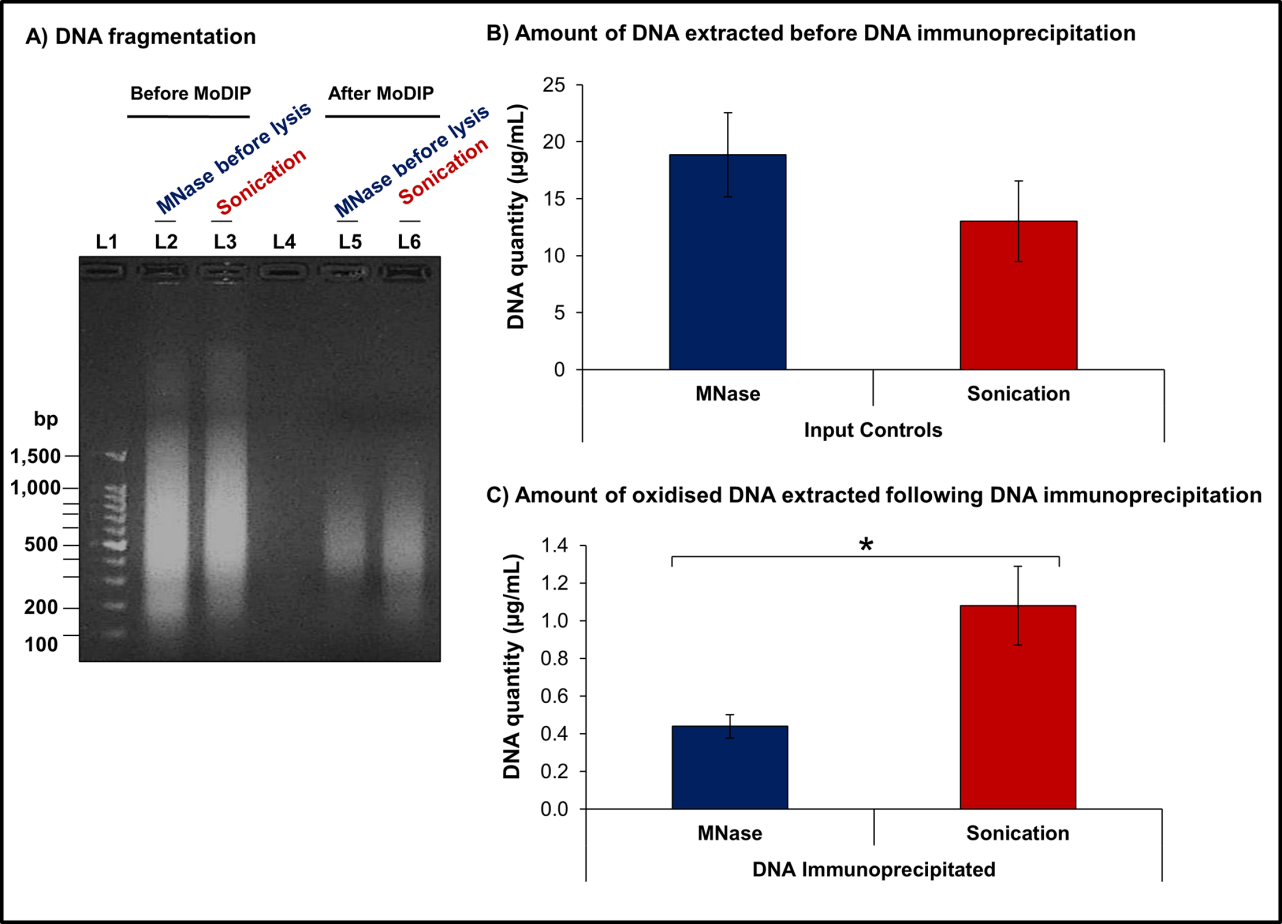
Quantification of DNA from human spermatozoa before and after modified immunoprecipitation to extract oxidised DNA. **a).** Resolution of isolated DNA via electrophoresis illustrated successful fragmentation was achieved using both experimental methods before and after MoDIP. Further, both methods extracted similar amounts of DNA with an equivalent size range before MoDIP. Following immunoprecipitation, method 2 (sonication) isolated comparatively more oxidised DNA. **L1:** 100bp DNA Ladder. **L2:** DNA fragments extracted using method 4 (MNase before cell lysis). **L3:** DNA fragments obtained following method 2 (sonication). **L4:** Empty Lane. **L5:** Oxidised DNA extracted following MoDIP and method 4 (MNase before cell lysis). **L6:** Oxidised DNA extracted following MoDIP and method 2 (sonication). **b)** The amount of DNA recovered from human spermatozoa before MoDIP was not significantly different between samples fragmented via sonication or MNase digestion. **c)** Significant increase in the amount of oxidised DNA extracted from sperm cells following MoDIP in samples fragmented via sonication (Mann-Whitney U test, p = 0.02, n = 4). *p<0.05.

Overall, we observed that both experimental approaches performed equally well, generating DNA fragments in the desired range of 200–1,000 bp before and after the MoDIP (Fig 6A). Interestingly, the amount of DNA extracted from cells prior to MoDIP was not significantly different (Mann-Whitney U test, p = 0.34, n = 4) following sonication or MNase digestion (Fig 6B), although on average there was slight decrease in the amount collected after sonication (13.0 ± 3.5 μg/mL) compared to the amount recovered from samples treated with MNase (18.9 ± 3.9 μg/mL). After the MoDIP, the amount of oxidised DNA extracted from cells was significantly higher (Mann-Whitney U test, p = 0.02, n = 4) in samples sheared via sonication than in those fragmented by MNase digestion (Fig 6C). Importantly, the amount of oxidised DNA extracted from identical samples of the same individual was found to significantly increase in samples following sonication rather than in samples submitted to MNase digestion, a result consistent with our previous observations (Fig 5B). Providing further evidence additional DNA oxidative damage can be inflicted by sonic vibration and thus demonstrating that methods using sonication should not be used in studies attempting to investigate the effects of oxidative damage to the DNA of cells.

### The bioinformatic framework successfully maps and clusters DNA sequences

The experimental protocols described in this study (Figs 1 and 2) aim to improve the existing techniques and provide the means to investigate the effects of oxidative damage to the genetic content of sperm cells. With this objective in mind, we designed an experimental approach that was able to extract exclusively oxidised DNA fragments from human spermatozoa, which we propose can be identified and characterised following next-generation sequencing and bioinformatic analysis. To start with, we were able to extract a large enough amount of genetic material from cells (18.9 ± 3.9 μg/mL), following steps described in methods 4 (MNase before cell lysis) and MoDIP, a value above the minimum threshold of detection (10 ng/mL) for standard next-generation sequencing.

While a completely independent study is required for the identification and characterisation of the genomic regions vulnerable to oxidative damage in human spermatozoa, we were able to develop and test a bioinformatic pipeline framework (Fig 3) in preparation of obtaining sequencing results. After assembling a mock dataset, the DNA sequences were submitted to multiple programs and algorithms to map the sequences against a reference genome, analyse their genetic composition and assemble them into continuous clusters.

The Bowtie short read alignment program was run first using the default parameters to map each DNA sequence in the mock dataset against the human reference genome. The alignment output from Bowtie was able to map to single locations 80% of the sequences, with the remaining 20% mapping to multiple genomic sites. Next, Bowtie ran with the–n alignment and–best mode options selected, restricting the number of mismatches allowed in the sequence, increase the number of sequences mapping to a single location to 89%. Comparing the mapped locations with the known genomic locations for each sequence in the dataset revealed that running Bowtie with stricter rules accurately mapped the sequences to their actual positions in the genome. Close examination of the sequences with ambiguous mapping showed that these sequences contained more 20 bases covering regions of repetitive DNA, and thus were too generic to accurately map to a single location of the genome. A modified mock dataset containing sequences with single point mutations, deletions and insertions was then processed by Bowtie 2, a more refined algorithm that permits to map gapped alignments that the original Bowtie program cannot process or accurately map. Bowtie 2 was then able to map to a single location 87% of the sequences in the mock dataset against the human reference genome. Comparison of the mapped and original genomic locations revealed that Bowtie 2 successfully recognised and aligned 85% of modified sequences. In 2% of the sequences aligned, the program incorrectly mapped the sequence to a genome position other that its original location, due to the insertion of 5 new bases that altered the sequence of nucleotides to create a new sequence motif. Since these modified sequences no longer closely matched the original sequences, Bowtie 2 achieved better alignments by matching them to alternative portions of the reference genome.

The sequences in the mock datasets were then analysed with the RepeatMasker program in conjunction with the Repbase library, to separately screen the successfully mapped and unaligned sequences for known human repeat elements and characterise their genomic composition. All unaligned sequences were shown to be composed almost exclusively of repetitive DNA, primarily SINEs, LINEs or microsatellites, and validating the difficulty that the alignment programs had into mapping these sequences was due to sequences not containing unique identifiable markers. Successfully mapped sequences were composed primarily of uniquely identifiable sequences of nucleotides (60% of all bases analysed) and repetitive DNA elements (40%). Due to the constructed nature of the dataset, the GC level content of the sequences was not taken in consideration at this time since sequences had not been randomly selected.

At this stage of the bioinformatic framework, sequencing data could no longer be analysed separately and individual sample results needed to be taken in context with results from controls. The negative control samples obtained by MoDIP, paired with samples treated with antibodies (Fig 6C), were found previously to contain only minute amounts of DNA, close to the detection threshold of quantifying assays (data not shown). This suggests that these extant sequences were a form of background noise associated with the experimental protocol that needed to be removed from the MoDIP results. For this purpose, we constructed our own Perl algorithm that took the sequences and their genetic coordinates in the negative control samples and parsed the positive results, first removing all sequences that had the same identity and location in both datasets, and then removed sequences that were either completely within or overlapped with the background noise sequences. The algorithm performed these calculations evaluating the chromosome, start and end positions and comparing them against the background dataset. Sequences that fulfilled all the established parameters were effectively removed from the dataset and excluded from further consideration. The accuracy of this constructed algorithm was tested by selecting and labelling a set of sequences from the mock dataset as background noise and examining the output generated after running the program with both set of data. As expected the program returned a list of sequences containing all but the designated background noise.

After adjusting the dataset to remove all background noise, the remaining sequences had to be investigated to determine whether they formed cluster of closely related sequences. Following MoDIP and DNA sequencing, such clusters of oxidised DNA could be used to calculate hotspots of oxidation and identify genomic regions vulnerable to oxidative stress. For this purpose, we constructed a Perl algorithm to sort through the DNA sequences and group together those sequences that overlapped each other. To increase the computational power and speed of the calculations, the algorithm was divided into two modules, the first separated the sequences by chromosome, then sorted the sequences by numerical order based on starting position and stored the data collected from each chromosome in a series of temporary files, so as to avoid keeping them in memory and slowing computation. The second module opened each of these temporary files and evaluated, based on start and end positions, whether consecutive sequences overlapped with each other. If so, the grouping added an extra sequence and expanded its boundaries to include newly acquired sequences until the next sequence failed to be in contact with the present group. After reaching the end of the cluster the program would count all the sequences within the cluster, close the group and start a new grouping starting with the first sequence outside the previous group. While developing the script, an option was introduced that allowed the calculation to consider consecutive sequences, that although demonstrating no overlap were within a defined number of base pairs away from each other, to be part of the same cluster. This option was coded into the program to allow for instances where close sequences were less than a sequence away from each other, an occurrence predicted to occur in real datasets due to sequencing errors or ambiguous mapping. After several rounds of optimisation, the developed algorithm was able to aggregate the sequences in the mock dataset to match the original sequence groupings, and generate a processed dataset that could be imported into a statistical software and analysed. However, due to the nature of the mock dataset used to test the bioinformatic pathway, while the statistical model could be developed, no significance could be interpreted from this analysing this data.

## Discussion

In this study, we showed that the combination of MNase digestion to generate DNA fragments and removal of oxidising and reducing compounds from cell lysis and DNA extraction processes improved significantly the experimental yield while inflicting the least amount of oxidative damage to DNA. Given the prevalence of oxidative damage in the DNA of mature spermatozoa of human males with reduced fertility [1–4], the development of an improved methodology for the extraction and fragmentation of genetic material from human spermatozoa was of critical importance to allow the investigation of the genomic consequences of oxidative stress in humans.

A common method used in the preparation of genetic samples before submitting the genetic material extracted from spermatozoa to ChIP and next-generation sequencing, involves the usage of sonication to achieve DNA fragmentation [29, 52, 53]. However, ultrasonic waves produce gaseous cavitation in liquid solutions that shear and break the DNA molecule through resonance vibration [54, 55]. Additionally, sonication can induce oxidative damage to the overall DNA structure causing the oxidation of nucleotides by increasing the number of free radicals that indirectly inflict oxidative damage to the DNA [54–56]. While the introduction of DNA oxidative damage may be of little consequence to studies investigating proteins bound to chromatin or aimed at detecting specific gene mutations, the damaged incurred from the fragmentation process has the potential to severely compromise studies attempting to investigate the consequences of oxidative stress. Therefore, we proposed that an alternative to sonication should be utilised to reduce the amount of experimentally-induced oxidation during sample preparation.

The enzymatic digestion of chromatin by the nucleases offered a viable alternative to sonication, particularly using MNase to fragment DNA. Although past studies have suggested that MNase digest the DNA preferentially at specific genomic locations [57–59], this apparent specificity has been linked to chromatin accessibility. With the arrangement of the chromatin structure determining which regions were exposed to nuclease, fragmentation was found to take place primarily at regions of the genome not bound by structural proteins and thus resulted in the generation of nucleosome sized fragments [59–61], strongly suggesting that chromatin relaxation remains a necessary step prior to MNase digestion.

In spermatozoa, treating the cells with the detergent Triton X-100 coupled with a reducing agent, either DTT or β-ME, is known to relax the chromatin structure. Interestingly, the presence of decondensed chromatin in live sperm has been classified as a form of DNA damage [62–64], that is known to lead to abnormal zygotic DNA synthesis after fertilisation [65, 66]. In the context of our experimental aims, the treatment was required to relax the chromatin structure as to provide MNase with unrestricted access to DNA, and since cell lysis was promoted immediately after digestion, these treated spermatozoa were obliterated and not used to fertilise an oocyte. Thus, this structural from of damage was of no consequence to the experimental results obtained. Nevertheless, these reducing agents are known to indirectly induce oxidative DNA damage. While DTT and β-ME have a low redox potential of -0.26V and -0.33V, respectively [67, 68], prolonged exposure at relatively high concentrations to these chemicals can induced DNA damage [62, 63]. Due to the unstable nature of these compounds and their tendency to facilitate oxidation of dissolved oxygen by electron donation during redox activity [67, 69, 70], ROS becomes more abundant and starts to inflict oxidative damage to the DNA in the cells.

With this goal in mind, compounds with highly reactive hydroxyl radicals (-OH), superoxide radicals (O_2_^-^) and oxidation catalysers were completely removed, or significantly reduced, from the existing protocols to prevent the generation of ROS and free radicals capable of inducing oxidative lesions. In experimental steps of existing protocols calling for the usage of β-ME, DTT was used instead due to its relative stability. Additionally, the concentration and the duration of the exposure to DTT was reduced to a level that minimised the induction of lesions but sufficient to break disulphide bonds and relax the overall chromatin structure [62–64]. Accordingly, our results from the modified protocols showed clear reduction in experimentally-induced collateral damage, particularly when substituting sonication with MNase digestion prior to cell lysis and recovery of DNA. An alternative method where MNase digestion took place after cell lysis and the isolation of DNA was developed and tested but the results demonstrated a comparative increase in the amount of oxidative damage. We propose that prolonged exposure of naked DNA to oxidative agents during the multiple rounds of phenol-chloroform extractions and losses inflicted over the course of successive washing steps provide a viable explanation for the observed reduction in yield and increased amount of oxidative lesion at the end of this experimental method.

Having determined the sensitivity of DNA experimentally-induced oxidative stresses following several modified protocols, we investigated the suitability of these methods in preparing the samples for an immunoprecipitation designed to extract oxidised DNA from sperm cells. Once more the results confirmed not only that MNase digestion provided a good alternative to sonication by extracting greater yields of DNA from the same number cells before the MoDIP, but also that the amount of oxidised DNA extracted following the MoDIP increased in sonicated samples. Additionally, the final quantities of DNA extracted from each of these protocols were found to be above the minimum threshold of detection for next-generation sequencing, suggesting that the genomic material from these samples could be successfully sequenced and characterised. In anticipation of obtaining such sequencing data from human sperm samples, we constructed a bioinformatics pipeline framework, using currently available software integrated with newly constructed algorithms, to successfully map the sequenced fragments of oxidised DNA to their correct genomic positions in the human genome and to calculate probable focal points of oxidation. Thus, providing a useful outline to investigate the burden of oxidative stress sperm cells, and although these procedures were designed for human cells, we predict no significant obstacles to adapt these protocols to other species with similar chromatin arrangements.

## Supporting information

S1 File parse_bowtie_alignments.pl. Perl script constructed to read and extract the mapped location of each read to the human reference genome (GRCh38/hg39), the read reference name and nucleotide sequence form the output files generated by the read alignment programs bowtie and bowtie 2.
(DOCX)

S2 File coverage.pl. Perl script constructed to calculate the read coverage by comparing the alignment positions of mapped reads against the human reference genome (GRCh38/hg39). Accuracy of the read alignment programs bowtie and bowtie 2 assessed from depth of coverage of individual base pairs.
(DOCX)

S3 File clean_backgroundNoise.pl. Perl algorithm developed to remove DNA sequences classified as background noise found in negative control samples from samples containing oxidised DNA fragments extracted by antibody during the MoDIP protocol. Script generates two files, one containing the all sequences without background noise and the other with all the removed sequences.
(DOCX)

S4 File clusterMaker.pl. Perl algorithm created to sort the mapped sequences of DNA from samples and group overlapping ones into clusters of continuous reads. The algorithm first sorts the reads by chromosome and location and then based on start and end positions of consecutive reads dynamically aggregate them into clusters. Script includes the option of adding the interval of base pairs (0-50bp) between non-overlapping sequences to still be considered part of the same grouping.
(DOCX)
