# Supplementary material for: Improved methods of DNA extraction from human spermatozoa that mitigate experimentally-induced oxidative DNA damage
Source: PLoS One. 2018 Mar 26;13(3):e0195003. doi: 10.1371/journal.pone.0195003 (PMC5868848; doi:10.1371/journal.pone.0195003)
Supplement: S1 File — Perl script constructed to read and extract the mapped location of each read to the human reference genome (GRCh38/hg39), the read reference name and nucleotide sequence form the output files generated by the read alignment programs bowtie and bowtie 2. (DOCX) [file pone.0195003.s001.docx]

**S1 File**. **parse_bowtie_alignments.pl** Perl script constructed to read and extract the mapped location of each read to the human reference genome (GRCh38/hg39), the read reference name and nucleotide sequence form the output files generated by the read alignment programs bowtie and bowtie 2.

#!/usr/local/bin/perl

$input_bowtie = $ARGV[0]; #name file containing bowtie aligments

open (IN1, "$input_bowtie") || die "Error: cannot open $input_bowtie.\n”; #open bowtie file

my @bowtie = (<IN1>); #Read file into array

$input_bowtie2 = $ARGV[1]; #name file containing bowtie2 aligments

open (IN2, "$input_bowtie2") || die "Error: cannot open $input_bowtie2.\n"; #open bowtie2 file

my @bowtie2 = (<IN2>); #Read file into array

#provide sample identifier to 1)specifically name output_file 2)identify the original sample of each read mapped

my $sample_ID = $ARGV[2];

$outputFile1 = "mapped_$sample_ID.txt"; #name the tab-delimited text output file

open (OUT, '>', $outputFile1) || die "Error: cannot locate $outputFile1.\n"; #open output file for writing

my $counter = 0; #initiate a simple counter to number each individual read.

foreach $line (@bowtie){ #read the bowtie alignment file line by line

#regular expression statement to identify and parse relevant information

if ($line =~ /^\w.+\t(\+||\-)\t(\w.+)\t(\d+)\t(\w+)\t/){

$counter++; #add another iteration of the counter

#$strand = $1; #strand information identified but not used.

$chr = $2; #chromosome ID

$start_position = $3; #start position in reference genome that corresponds to mapped read

$sequence = $4; #sequence of nucleotides aligned to reference genome

$end_position = $start_position + length($sequence);

#calculate end position from start position and the length of the mapped sequence

#print relevant information to file

print OUT "$sample_ID\_$counter\t$chr:$start_position-$end_position\t$sequence\n";

}

}

foreach $lines (@bowtie2){ #read the bowtie alignment file line by line

#regular expression statement to identify and parse relevant information

if ($lines =~ /^\w.+\t\d+\t(\w.+)\t(\d+)\t\d+\t\d.+\t.\t\d+\t\d+\t(\w+)\t/){

$counter++; #add another iteration of the counter

$chr_bwt2 = $1; #chromosome ID

$start_po_bwt2 = $2; #start position in reference genome that corresponds to mapped read

$sequence_bwt2 = $3; #sequence of nucleotides aligned to reference genome

$end_po_bwt2 = $start_po_bwt2 + length($sequence_bwt2);

#calculate end position from start position and the length of the mapped sequence

#print relevant information to file

print OUT "$sample_ID\_$counter\t$chr_bwt2:$start_po_bwt2-$end_po_bwt2\t $sequence_bwt2\n";

}

}

#close all files

close (IN1); close (IN2); close(OUT);

print "Script Complete\n"; #terminate script
