# Supplementary material for: Improved methods of DNA extraction from human spermatozoa that mitigate experimentally-induced oxidative DNA damage
Source: PLoS One. 2018 Mar 26;13(3):e0195003. doi: 10.1371/journal.pone.0195003 (PMC5868848; doi:10.1371/journal.pone.0195003)
Supplement: S2 File — Perl script constructed to calculate the read coverage by comparing the alignment positions of mapped reads against the human reference genome (GRCh38/hg39). Accuracy of the read alignment programs bowtie and bowtie 2 assessed from depth of coverage of individual base pairs. (DOCX) [file pone.0195003.s002.docx]

**S2 File. coverage.pl** Perl script constructed to calculate the read coverage by comparing the alignment positions of mapped reads against the human reference genome (GRCh38/hg39). Accuracy of the read alignment programs bowtie and bowtie 2 assessed from depth of coverage of individual base pairs.

#!/usr/local/bin/perl

##Before running script the files with the mapped reads were sorted using the linux command

## >sort mapped_sample01.txt -k 2 > mapped_sample01_sorted.txt

$inputFile = $ARGV[0]; #name file containing sorted mapped reads

open (IN, "$inputFile") || die "Error: cannot open $inputFile.\n"; #open sorted reads file

my @mappedReads = (<IN>); #Read file into array

$sample_ID = $ARGV[1]; #provide sample identifier to specifically name output_file

$outputFile = "coverage_$sample_ID.bed"; #name the tab-delimited text output file

open (OUT, '>', $outputFile)|| die "Error: cannot locate $outputFile.\n"; #open output file for writing

#print header according to .bed format into outputfile

print OUT "browser position chr1:1-248956422\n"; #determines intial window position on UCSC Genome browser

print OUT "browser hide all\n"; #hide all non-relevant information on browser

print OUT "browser pack refGene encodeRegions\n"; #display pack refGene track on browser

print OUT "browser full altGraph\n"; #specify bedGraph subformat

#specify track name, description, colour and priority

print OUT "track type=bedGraph name=\"Coverage $sample_ID\" description=\"Coverage $sample_ID\" visibility=full color=200,100,0 altColor=0,100,200 priority=1";

print OUT "\n"; #change line before writting individual bp frequencies

#initiate variables

my $chr1_bp; my $chr2_bp; my $chr3_bp; my $chr4_bp; my $chr5_bp; my $chr6_bp;

my $chr7_bp; my $chr8_bp; my $chr9_bp; my $chr10_bp; my $chr11_bp; my $chr12_bp;

my $chr13_bp; my $chr14_bp; my $chr15_bp; my $chr16_bp; my $chr17_bp;

my $chr18_bp; my $chr19_bp; my $chr20_bp; my $chr21_bp; my $chr22_bp; my $chrX_bp; my $chrY_bp;

foreach $chr1_bp (1..248956422){ #iterate for each individual bp on chr1, one base pair at a time

my $count = 0; #initiate counter for each bp

foreach $read (@mappedReads){ #read mapped reads file line by line

if ($read =~ /^(\d.+)\t(\w.+)\:(\d+)-(\d+)\t(\w+)/) { #regular expression statement to identify and parse relevant information

#$ID = $1; #ID information identified but not used.

$chr = $2; #chromosome ID

$startPOS = $3; #start position of read on reference genome

$endPOS = $4; #end position of read on reference genome

#$seq = $5; #sequence identified but not used.

if ($chr eq "chr1") { #conditional statement to only select reads from chr1

if ($chr1_bp >= $startPOS && $chr1_bp <= $endPOS) {

$count++; #count number of reads that cover the individual bp in the loop based on start and end positions of each read

}

if ($chr1_bp > $endPOS) { #if the bp in greater than the end position of a read

next; #move to the next read

}

}

else { #if read maps to chr other than chr1

next; #move to next read

}

}

}

#after all reads that cover each bp were counted print to file

if ($count > 0) { #positions with no cover ignored to reduce file size

$posSTART = $chr1_bp; #identify bp position

$posEND = $chr1_bp + 1; #add 1 to bp position because browser cannot handle equal positions for start and end

print OUT "chr1\t$posSTART\t$posEND\t$count\n"; #print relevant information to file

}

}

#similar loops below perform identical function for chr2 to chr Y, only the number of bp in each chr bp array change to match chr sizes

foreach $chr2_bp (1..242193529){

my $count = 0;

foreach $read (@mappedReads){

if ($read =~ /^(\d.+)\t(\w.+)\:(\d+)-(\d+)\t(\w+)/) {

#$ID = $1;

$chr = $2;

$startPOS = $3;

$endPOS = $4;

#$seq = $5;

if ($chr eq "chr2") {

if ($chr2_bp >= $startPOS && $chr2_bp <= $endPOS) {

$count++;

}

if ($chr2_bp > $endPOS) {

next;

}

}

else {

next;

}

}

}

if ($count > 0) {

$posSTART = $chr2_bp;

$posEND = $chr2_bp + 1;

print OUT "chr2\t$posSTART\t$posEND\t$count\n";

}

}

foreach $chr3_bp (1..198295559){

my $count = 0;

foreach $read (@mappedReads){

if ($read =~ /^(\d.+)\t(\w.+)\:(\d+)-(\d+)\t(\w+)/) {

#$ID = $1;

$chr = $2;

$startPOS = $3;

$endPOS = $4;

#$seq = $5;

if ($chr eq "chr3") {

if ($chr3_bp >= $startPOS && $chr3_bp <= $endPOS) {

$count++;

}

if ($chr3_bp > $endPOS) {

next;

}

}

else {

next;

}

}

}

if ($count > 0) {

$posSTART = $chr3_bp;

$posEND = $chr3_bp + 1;

print OUT "chr3\t$posSTART\t$posEND\t$count\n";

}

}

foreach $chr4_bp (1..190214555){

my $count = 0;

foreach $read (@mappedReads){

if ($read =~ /^(\d.+)\t(\w.+)\:(\d+)-(\d+)\t(\w+)/) {

#$ID = $1;

$chr = $2;

$startPOS = $3;

$endPOS = $4;

#$seq = $5;

if ($chr eq "chr4") {

if ($chr4_bp >= $startPOS && $chr4_bp <= $endPOS) {

$count++;

}

if ($chr4_bp > $endPOS) {

next;

}

}

else {

next;

}

}

}

if ($count > 0) {

$posSTART = $chr4_bp;

$posEND = $chr4_bp + 1;

print OUT "chr4\t$posSTART\t$posEND\t$count\n";

}

}

foreach $chr5_bp (1..181538259){

my $count = 0;

foreach $read (@mappedReads){

if ($read =~ /^(\d.+)\t(\w.+)\:(\d+)-(\d+)\t(\w+)/) {

#$ID = $1;

$chr = $2;

$startPOS = $3;

$endPOS = $4;

#$seq = $5;

if ($chr eq "chr5") {

if ($chr5_bp >= $startPOS && $chr5_bp <= $endPOS) {

$count++;

}

if ($chr5_bp > $endPOS) {

next;

}

}

else {

next;

}

}

}

if ($count > 0) {

$posSTART = $chr5_bp;

$posEND = $chr5_bp + 1;

print OUT "chr5\t$posSTART\t$posEND\t$count\n";

}

}

foreach $chr6_bp (1..170805979){

my $count = 0;

foreach $read (@mappedReads){

if ($read =~ /^(\d.+)\t(\w.+)\:(\d+)-(\d+)\t(\w+)/) {

#$ID = $1;

$chr = $2;

$startPOS = $3;

$endPOS = $4;

#$seq = $5;

if ($chr eq "chr6") {

if ($chr6_bp >= $startPOS && $chr6_bp <= $endPOS) {

$count++;

}

if ($chr6_bp > $endPOS) {

next;

}

}

else {

next;

}

}

}

if ($count > 0) {

$posSTART = $chr6_bp;

$posEND = $chr6_bp + 1;

print OUT "chr6\t$posSTART\t$posEND\t$count\n";

}

}

foreach $chr7_bp (1..159345973){

my $count = 0;

foreach $read (@mappedReads){

if ($read =~ /^(\d.+)\t(\w.+)\:(\d+)-(\d+)\t(\w+)/) {

#$ID = $1;

$chr = $2;

$startPOS = $3;

$endPOS = $4;

#$seq = $5;

if ($chr eq "chr7") {

if ($chr7_bp >= $startPOS && $chr7_bp <= $endPOS) {

$count++;

}

if ($chr7_bp > $endPOS) {

next;

}

}

else {

next;

}

}

}

if ($count > 0) {

$posSTART = $chr7_bp;

$posEND = $chr7_bp + 1;

print OUT "chr7\t$posSTART\t$posEND\t$count\n";

}

}

foreach $chr8_bp (1..145138636){

my $count = 0;

foreach $read (@mappedReads){

if ($read =~ /^(\d.+)\t(\w.+)\:(\d+)-(\d+)\t(\w+)/) {

#$ID = $1;

$chr = $2;

$startPOS = $3;

$endPOS = $4;

#$seq = $5;

if ($chr eq "chr8") {

if ($chr8_bp >= $startPOS && $chr8_bp <= $endPOS) {

$count++;

}

if ($chr8_bp > $endPOS) {

next;

}

}

else {

next;

}

}

}

if ($count > 0) {

$posSTART = $chr8_bp;

$posEND = $chr8_bp + 1;

print OUT "chr8\t$posSTART\t$posEND\t$count\n";

}

}

foreach $chr9_bp (1..138394717){

my $count = 0;

foreach $read (@mappedReads){

if ($read =~ /^(\d.+)\t(\w.+)\:(\d+)-(\d+)\t(\w+)/) {

#$ID = $1;

$chr = $2;

$startPOS = $3;

$endPOS = $4;

#$seq = $5;

if ($chr eq "chr9") {

if ($chr9_bp >= $startPOS && $chr9_bp <= $endPOS) {

$count++;

}

if ($chr9_bp > $endPOS) {

next;

}

}

else {

next;

}

}

}

if ($count > 0) {

$posSTART = $chr9_bp;

$posEND = $chr9_bp + 1;

print OUT "chr9\t$posSTART\t$posEND\t$count\n";

}

}

foreach $chr10_bp (1..133797422){

my $count = 0;

foreach $read (@mappedReads){

if ($read =~ /^(\d.+)\t(\w.+)\:(\d+)-(\d+)\t(\w+)/) {

#$ID = $1;

$chr = $2;

$startPOS = $3;

$endPOS = $4;

#$seq = $5;

if ($chr eq "chr10") {

if ($chr10_bp >= $startPOS && $chr10_bp <= $endPOS) {

$count++;

}

if ($chr10_bp > $endPOS) {

next;

}

}

else {

next;

}

}

}

if ($count > 0) {

$posSTART = $chr10_bp;

$posEND = $chr10_bp + 1;

print OUT "chr10\t$posSTART\t$posEND\t$count\n";

}

}

foreach $chr11_bp (1..135086622){

my $count = 0;

foreach $read (@mappedReads){

if ($read =~ /^(\d.+)\t(\w.+)\:(\d+)-(\d+)\t(\w+)/) {

#$ID = $1;

$chr = $2;

$startPOS = $3;

$endPOS = $4;

#$seq = $5;

if ($chr eq "chr11") {

if ($chr11_bp >= $startPOS && $chr11_bp <= $endPOS) {

$count++;

}

if ($chr11_bp > $endPOS) {

next;

}

}

else {

next;

}

}

}

if ($count > 0) {

$posSTART = $chr11_bp;

$posEND = $chr11_bp + 1;

print OUT "chr11\t$posSTART\t$posEND\t$count\n";

}

}

foreach $chr12_bp (1..133275309){

my $count = 0;

foreach $read (@mappedReads){

if ($read =~ /^(\d.+)\t(\w.+)\:(\d+)-(\d+)\t(\w+)/) {

#$ID = $1;

$chr = $2;

$startPOS = $3;

$endPOS = $4;

#$seq = $5;

if ($chr eq "chr12") {

if ($chr12_bp >= $startPOS && $chr12_bp <= $endPOS) {

$count++;

}

if ($chr12_bp > $endPOS) {

next;

}

}

else {

next;

}

}

}

if ($count > 0) {

$posSTART = $chr12_bp;

$posEND = $chr12_bp + 1;

print OUT "chr12\t$posSTART\t$posEND\t$count\n";

}

}

foreach $chr13_bp (1..114364328){

my $count = 0;

foreach $read (@mappedReads){

if ($read =~ /^(\d.+)\t(\w.+)\:(\d+)-(\d+)\t(\w+)/) {

#$ID = $1;

$chr = $2;

$startPOS = $3;

$endPOS = $4;

#$seq = $5;

if ($chr eq "chr13") {

if ($chr13_bp >= $startPOS && $chr13_bp <= $endPOS) {

$count++;

}

if ($chr13_bp > $endPOS) {

next;

}

}

else {

next;

}

}

}

if ($count > 0) {

$posSTART = $chr13_bp;

$posEND = $chr13_bp + 1;

print OUT "chr13\t$posSTART\t$posEND\t$count\n";

}

}

foreach $chr14_bp (1..107043718){

my $count = 0;

foreach $read (@mappedReads){

if ($read =~ /^(\d.+)\t(\w.+)\:(\d+)-(\d+)\t(\w+)/) {

#$ID = $1;

$chr = $2;

$startPOS = $3;

$endPOS = $4;

#$seq = $5;

if ($chr eq "chr14") {

if ($chr14_bp >= $startPOS && $chr14_bp <= $endPOS) {

$count++;

}

if ($chr14_bp > $endPOS) {

next;

}

}

else {

next;

}

}

}

if ($count > 0) {

$posSTART = $chr14_bp;

$posEND = $chr14_bp + 1;

print OUT "chr14\t$posSTART\t$posEND\t$count\n";

}

}

foreach $chr15_bp (1..101991189){

my $count = 0;

foreach $read (@mappedReads){

if ($read =~ /^(\d.+)\t(\w.+)\:(\d+)-(\d+)\t(\w+)/) {

#$ID = $1;

$chr = $2;

$startPOS = $3;

$endPOS = $4;

#$seq = $5;

if ($chr eq "chr15") {

if ($chr15_bp >= $startPOS && $chr15_bp <= $endPOS) {

$count++;

}

if ($chr15_bp > $endPOS) {

next;

}

}

else {

next;

}

}

}

if ($count > 0) {

$posSTART = $chr15_bp;

$posEND = $chr15_bp + 1;

print OUT "chr15\t$posSTART\t$posEND\t$count\n";

}

}

foreach $chr16_bp (1..90338345){

my $count = 0;

foreach $read (@mappedReads){

if ($read =~ /^(\d.+)\t(\w.+)\:(\d+)-(\d+)\t(\w+)/) {

#$ID = $1;

$chr = $2;

$startPOS = $3;

$endPOS = $4;

#$seq = $5;

if ($chr eq "chr16") {

if ($chr16_bp >= $startPOS && $chr16_bp <= $endPOS) {

$count++;

}

if ($chr16_bp > $endPOS) {

next;

}

}

else {

next;

}

}

}

if ($count > 0) {

$posSTART = $chr16_bp;

$posEND = $chr16_bp + 1;

print OUT "chr16\t$posSTART\t$posEND\t$count\n";

}

}

foreach $chr17_bp (1..83257441){

my $count = 0;

foreach $read (@mappedReads){

if ($read =~ /^(\d.+)\t(\w.+)\:(\d+)-(\d+)\t(\w+)/) {

#$ID = $1;

$chr = $2;

$startPOS = $3;

$endPOS = $4;

#$seq = $5;

if ($chr eq "chr17") {

if ($chr17_bp >= $startPOS && $chr17_bp <= $endPOS) {

$count++;

}

if ($chr17_bp > $endPOS) {

next;

}

}

else {

next;

}

}

}

if ($count > 0) {

$posSTART = $chr17_bp;

$posEND = $chr17_bp + 1;

print OUT "chr17\t$posSTART\t$posEND\t$count\n";

}

}

foreach $chr18_bp (1..80373285){

my $count = 0;

foreach $read (@mappedReads){

if ($read =~ /^(\d.+)\t(\w.+)\:(\d+)-(\d+)\t(\w+)/) {

#$ID = $1;

$chr = $2;

$startPOS = $3;

$endPOS = $4;

#$seq = $5;

if ($chr eq "chr18") {

if ($chr18_bp >= $startPOS && $chr18_bp <= $endPOS) {

$count++;

}

if ($chr18_bp > $endPOS) {

next;

}

}

else {

next;

}

}

}

if ($count > 0) {

$posSTART = $chr18_bp;

$posEND = $chr18_bp + 1;

print OUT "chr18\t$posSTART\t$posEND\t$count\n";

}

}

foreach $chr19_bp (1..58617616){

my $count = 0;

foreach $read (@mappedReads){

if ($read =~ /^(\d.+)\t(\w.+)\:(\d+)-(\d+)\t(\w+)/) {

#$ID = $1;

$chr = $2;

$startPOS = $3;

$endPOS = $4;

#$seq = $5;

if ($chr eq "chr19") {

if ($chr19_bp >= $startPOS && $chr19_bp <= $endPOS) {

$count++;

}

if ($chr19_bp > $endPOS) {

next;

}

}

else {

next;

}

}

}

if ($count > 0) {

$posSTART = $chr19_bp;

$posEND = $chr19_bp + 1;

print OUT "chr19\t$posSTART\t$posEND\t$count\n";

}

}

foreach $chr20_bp (1..64444167){

my $count = 0;

foreach $read (@mappedReads){

if ($read =~ /^(\d.+)\t(\w.+)\:(\d+)-(\d+)\t(\w+)/) {

#$ID = $1;

$chr = $2;

$startPOS = $3;

$endPOS = $4;

#$seq = $5;

if ($chr eq "chr20") {

if ($chr20_bp >= $startPOS && $chr20_bp <= $endPOS) {

$count++;

}

if ($chr20_bp > $endPOS) {

next;

}

}

else {

next;

}

}

}

if ($count > 0) {

$posSTART = $chr20_bp;

$posEND = $chr20_bp + 1;

print OUT "chr20\t$posSTART\t$posEND\t$count\n";

}

}

foreach $chr21_bp (1..46709983){

my $count = 0;

foreach $read (@mappedReads){

if ($read =~ /^(\d.+)\t(\w.+)\:(\d+)-(\d+)\t(\w+)/) {

#$ID = $1;

$chr = $2;

$startPOS = $3;

$endPOS = $4;

#$seq = $5;

if ($chr eq "chr21") {

if ($chr21_bp >= $startPOS && $chr21_bp <= $endPOS) {

$count++;

}

if ($chr21_bp > $endPOS) {

next;

}

}

else {

next;

}

}

}

if ($count > 0) {

$posSTART = $chr21_bp;

$posEND = $chr21_bp + 1;

print OUT "chr21\t$posSTART\t$posEND\t$count\n";

}

}

foreach $chr22_bp (1..50818468){

my $count = 0;

foreach $read (@mappedReads){

if ($read =~ /^(\d.+)\t(\w.+)\:(\d+)-(\d+)\t(\w+)/) {

#$ID = $1;

$chr = $2;

$startPOS = $3;

$endPOS = $4;

#$seq = $5;

if ($chr eq "chr22") {

if ($chr22_bp >= $startPOS && $chr22_bp <= $endPOS) {

$count++;

}

if ($chr22_bp > $endPOS) {

next;

}

}

else {

next;

}

}

}

if ($count > 0) {

$posSTART = $chr22_bp;

$posEND = $chr22_bp + 1;

print OUT "chr22\t$posSTART\t$posEND\t$count\n";

}

}

foreach $chrX_bp (1..156040895){

my $count = 0;

foreach $read (@mappedReads){

if ($read =~ /^(\d.+)\t(\w.+)\:(\d+)-(\d+)\t(\w+)/) {

#$ID = $1;

$chr = $2;

$startPOS = $3;

$endPOS = $4;

#$seq = $5;

if ($chr eq "chrX") {

if ($chrX_bp >= $startPOS && $chrX_bp <= $endPOS) {

$count++;

}

if ($chrX_bp > $endPOS) {

next;

}

}

else {

next;

}

}

}

if ($count > 0) {

$posSTART = $chrX_bp;

$posEND = $chrX_bp + 1;

print OUT "chrX\t$posSTART\t$posEND\t$count\n";

}

}

foreach $chrY_bp (1..57227415){

my $count = 0;

foreach $read (@mappedReads){

if ($read =~ /^(\d.+)\t(\w.+)\:(\d+)-(\d+)\t(\w+)/) {

#$ID = $1;

$chr = $2;

$startPOS = $3;

$endPOS = $4;

#$seq = $5;

if ($chr eq "chrY") {

if ($chrY_bp >= $startPOS && $chrY_bp <= $endPOS) {

$count++;

}

if ($chrY_bp > $endPOS) {

next;

}

}

else {

next;

}

}

}

if ($count > 0) {

$posSTART = $chrY_bp;

$posEND = $chrY_bp + 1;

print OUT "chrY\t$posSTART\t$posEND\t$count\n";

}

}

#close all files

close (IN); close(OUT);

print "Script Complete\n"; #terminate script
