# Supplementary material for: Improved methods of DNA extraction from human spermatozoa that mitigate experimentally-induced oxidative DNA damage
Source: PLoS One. 2018 Mar 26;13(3):e0195003. doi: 10.1371/journal.pone.0195003 (PMC5868848; doi:10.1371/journal.pone.0195003)
Supplement: S3 File — Perl algorithm developed to remove DNA sequences classified as background noise found in negative control samples from samples containing oxidised DNA fragments extracted by antibody during the MoDIP protocol. Script generates two files, one containing the all sequences without background noise and the other with all the removed sequences. (DOCX) [file pone.0195003.s003.docx]

**S3 File.** **clean_backgroundNoise.pl** Perl algorithm developed to remove DNA sequences classified as background noise found in negative control samples from samples containing oxidised DNA fragments extracted by antibody during the MoDIP protocol. Script generates two files, one containing the all sequences without background noise and the other with all the removed sequences.

#!/usr/local/bin/perl

##command variables

$no_antibody_sample = $ARGV[0]; #background noise read file i.e. MoDIP samples with no antibody

$antibody_sample = $ARGV[1]; #sample file with reads that need to be cleaned i.e. MoDIP samples with antibody

####Part 1#################################################################

##colapse identical reads into a single reads considered background noise###############

open (IN1, "$no_antibody_sample") || die "Error: cannot open $no_antibody_sample.\n"; #open background noise file

my @noise = (<IN1>); #Read file into array

$temp_File = "background_noise.tmp"; #name temporary file contained colapsed noise reads

open (OUT1, '>', $temp_File) || die "Error: cannot locate $temp_File.\n"; #open output file for writing

my @Seqs; #initiate array

foreach $background (@noise){ #read background noise file line by line

if ($background =~ /^(\d.+)\t((\w.+)\:(\d+)-(\d+))\t(\w+)/){ #regular expression statement to identify and parse relevant information

#$ID = $1; #ID information identified but not used.

$read_location = $2; #complete read information chr:startPOS-endPOS used to identify each read.

#$chr = $3; #chr information identified but not used.

#$start_POS = $4; #start position information identified but not used.

#$end_POS = $5; #end position identified but not used.

#$seq = $6; #sequence identified but not used.

push (@Seqs, $read_location); #add each individual read location into an array to be sorted

}

}

#sort all read and count duplicates. finish by printing each unique read location followed by number of identifical reads

my %hash;

$hash{$_}++ foreach (@Seqs);

print OUT1 "$_\t$hash{$_}\n" foreach (sort {$hash{$a} <=> $hash{$b}} keys %hash);

print OUT1 "END\n"; #add "END" as the last line of the file. Used later to know when finished reading the file.

#close all files

close (IN1);

close (OUT1);

print "Part 1 of Script Complete\n"; #inform usr that the first set of instructions have been completed

###Part 2###################################################################

##remove background noise from samples that will be used for clustering###############

open (IN2, "$antibody_sample") || die "Error: cannot open $antibody_sample.\n"; #open file to clean

my @reads = (<IN2>); #Read file into array

open (IN3, "$temp_File") || die "Error: cannot open $temp_File.\n"; #open file created in part 1 of the script (collapsed noise reads)

my @back_noise = (<IN3>); #Read file into array

$outputFile1 = "removed_reads_$antibody_sample"; #name file that will store reads that match or partial match background noise

open (OUT2, '>', $outputFile1) || die "Error: cannot locate $outputFile1.\n"; #open output file for writing

$outputFile2 = "clean_$antibody_sample"; #name clean file, that no longer contains background noise reads

open (OUT3, '>', $outputFile2) || die "Error: cannot locate $outputFile2.\n"; #open output file for writing

foreach $read (@reads){ #read file to clean line by line

if ($read =~ /^(\d.+)\t((\w.+)\:(\d+)-(\d+))\t(\w+)/){ #regular expression statement to identify and parse relevant information

#$ID = $1; #ID information identified but not used.

#$read_location = $2; #information identified but not used.

$chrNo = $3; #chromosome ID of read in file to clean

$start_POS = $4; #start position of read in file to clean

$end_POS = $5; #end position of read in file to clean

$line = $read; #store entire read line for later use

foreach $bc_noise (@back_noise) { #read background noise file one read at the time, start to finish in every loop

if ($bc_noise =~ /^(\w.+)\:(\d+)-(\d+)\t(\d+)/){ #regular expression statement to identify and parse relevant information

$noise_chrNo = $1; #chromosome ID of noise reads

$noise_start_POS = $2; #start position of noise reads

$noise_end_POS = $3; #end position of noise reads

#$freq = $4; #no. of duplicate noise reads information not used.

if ($chrNo eq $noise_chrNo){ #identify noise reads that belong to the same chromosome to reads to clean

$half_read_length = ($end_POS - $start_POS)/2; #calculate the midpoint of each read, will be used to find overlapping reads

$overlap_start = $end_POS - $half_read_length; #calculate the adjusted end_position of reads to determine if there is overlap with noise read

$overlap_end = $start_POS + $half_read_length; #calculate the adjusted start_position of reads to determine if there is overlap with noise read

if ($start_POS >= $noise_start_POS && $end_POS <= $noise_end_POS){ #catch all reads that are identical or completely within to background noise

print OUT2 $line; #print reads that match criteria to removed_reads file

last; #exit loop and move on to the next read.

}

elsif ($start_POS >= $noise_start_POS && $overlap_start <= $noise_end_POS){#catch reads that have at least 50% overlap on start of read with noise read

print OUT2 $line; #print reads that match criteria to removed_reads file

last; #exit loop and move on to the next read.

}

elsif ($overlap_end >= $noise_start_POS && $end_POS <= $noise_end_POS) {#catch reads that have at least 50% overlap on end of read with noise read

print OUT2 $line; #print reads that match criteria to removed_reads file

last; #exit loop and move on to the next read.

}

}

}

if ($bc_noise =~ /^END/){ #if the sample read does not match any of the criteria for removal and reaches the end of the background noise file then

print OUT3 $line; #print reads to clean file

}

}

}

}

#close all files

close (IN2);

close (IN3);

close (OUT2);

close (OUT3);

print "Script Complete\n"; #terminate script
