# Supplementary material for: Improved methods of DNA extraction from human spermatozoa that mitigate experimentally-induced oxidative DNA damage
Source: PLoS One. 2018 Mar 26;13(3):e0195003. doi: 10.1371/journal.pone.0195003 (PMC5868848; doi:10.1371/journal.pone.0195003)
Supplement: S4 File — Perl algorithm created to sort the mapped sequences of DNA from samples and group overlapping ones into clusters of continuous reads. The algorithm first sorts the reads by chromosome and location and then based on start and end positions of consecutive reads dynamically aggregate them into clusters. Script includes the option of adding the interval of base pairs (0-50bp) between non-overlapping sequences to still be considered part of the same grouping. (DOCX) [file pone.0195003.s004.docx]

**S4 File. clusterMaker.pl** Perl algorithm created to sort the mapped sequences of DNA from samples and group overlapping ones into clusters of continuous reads. The algorithm first sorts the reads by chromosome and location and then based on start and end positions of consecutive reads dynamically aggregate them into clusters. Script includes the option of adding the interval of base pairs (0-50bp) between non-overlapping sequences to still be considered part of the same grouping.

#!/usr/local/bin/perl

##command variables

$cleanedreads = $ARGV[0]; #clean file after noise removal

$interval = $ARGV[1]; #select number of base pairs that are allowed between continuous reads to join clusters

$sample_ID = $ARGV[2]; #provide sample ID to name files accordingly

####Part 1##################################################################

##Prepare clean read files by separating reads by individual chromosomes.##############

open (IN1, "$cleanedreads") || die "Error: cannot open $cleanedreads.\n"; #open clean reads file

@Map_reads = <IN1>; #Read file into array

#create array with all chromosome names

my @chromosomes = ('chr1', 'chr2', 'chr3', 'chr4', 'chr5', 'chr6', 'chr7', 'chr8', 'chr9',

'chr10', 'chr11', 'chr12', 'chr13', 'chr14', 'chr15', 'chr16', 'chr17',

'chr18', 'chr19','chr20', 'chr21', 'chr22', 'chrX', 'chrY');

foreach $chromosome (@chromosomes){ #iterate for each individual chromosome, one at a time

foreach $map_read (@Map_reads){ #read clean reads file line by line

if ($map_read =~ /^(\d.+)\t(\w.+)\:(\d+)-(\d+)\t(\w+)/){ #regular expression statement to identify and parse relevant information

$chr = $2; #chromosome No of each read in file

if ($chromosome eq $chr) { #if the chromosome number of each read matches the chromosome iteration then

$output = "Reads_in_$chromosome\_$sample_ID.temp"; #create output file name to store reads by chr

open (OUT, '>>', $output) || die "Error: cannot locate $output.\n"; #open output file for writing

print OUT $map_read; #print reads to file

close (OUT); #close output file

}

}

}

}

foreach $chromosome (@chromosomes){ #iterate for each individual chromosome, one at a time

$output = "Reads_in_$chromosome\_$sample_ID.temp"; #name which output file name to open

open (OUT, '>>', $output) || die "Error: cannot locate $output.\n"; #open output file for writing

print OUT "END\n"; #add "END" as the last line of the file. Used later to know when finished reading the file.

close (OUT); #close output file

}

print "Part 1 of Script Complete\n"; #inform usr that the first set of instructions have been completed

###Part 2###################################################################

##create cluster of reads. continuous/overlapping reads form individual clusters,

##allows interval of x bp between reads to be adjusted to permit short spaces between reads, interval values tested 0 to 50###################################################

foreach $chr (@chromosomes){ #iterate for each individual chromosome, one at a time

$inputFile = "Reads_in_$chr\_$sample_ID.temp"; #name file containing reads to be clustered

open (IN2, "$inputFile") || die "Error: cannot open $inputFile.\n"; #open reads file

@reads = <IN2>; #Read file into array

$outputFile1 = "All_clusters_Info_$interval\_$sample_ID.txt"; #name output that will hold all cluster information

open (OUT1, '>>', $outputFile1) || die "Error: cannot locate $outputFile1.\n"; #open output file for writing

$outputFile2 = "clustersFreq_$sample_ID.txt"; #name output that will hold clusters and frequency values

open (OUT2, '>>', $outputFile2) || die "Error: cannot locate $outputFile2.\n"; #open output file for writing

#print headers with column names to each output file

print OUT1 "Cluster_no\tStart_Position\tEnd_Position\tCluster_Size\tElements_in_Clusters\tElements_IDs\n";

print OUT2 "Cluster\tNo_Reads_in_Clusters\n";

foreach $read (@reads){ #read clean reads seperated by chromosome, line by line

#initiate variables;

my $ID;

my $startP;

my $endP;

if ($read =~ /^(\d.+)\t(\w.+)\:(\d+)-(\d+)\t(\w+)/){ #regular expression statement to identify and parse relevant information

$ID = $1; #individual reads ID number

$startP = $3; #start position of each read

$endP = $4; #end position of each read

clusterM($ID, $startP, $endP); #call cluster making fuction and pass variables to fuction

}

if ($read =~ /(END)/){ #at the end of the clean reads seperated by chromosome file

$ID = "END";

clusterM($ID); #call cluster maker fuction one last time to pass the final variables and flush the final cluster

}

}

#initiate function variables

my @IDs;

my $cStart = 0;

my $cEnd = 1;

my $counterNo = 0;

my $counterN = 0;

sub clusterM{ #cluster making function

my ($ID, $start1, $end1) = @_; #receive and transfer variables for read file

if ($ID == "END") { #flush the final cluster

$clustersize = $cEnd - $cStart; #calculate size of each cluster from cluster start and end positions

$elements = scalar@IDs - 1; #calculate number of reads in each cluster

print OUT1 "$counterNo\t$cStart\t$cEnd\t$clustersize\t$elements\t@IDs\n"; #print all cluster information to file

print OUT2 "$chr:$cStart-$cEnd\t$elements\n"; #print cluster ID and number of reads in each cluster to file

}

if ($start1 > $cEnd+$interval) { #calculate primary boundary of each cluster

if ($counterNo eq $counterN+1) { #if the previous loop closed cluster, calculate the size and number of reads in each cluster

$clustersize = $cEnd - $cStart;

$elements = scalar@IDs - 1;

print OUT1 "$counterNo\t$cStart\t$cEnd\t$clustersize\t$elements\t@IDs\n"; #print all cluster information to file

print OUT2 "$chr:$cStart-$cEnd\t$elements\n"; #print cluster ID and number of reads in each cluster to file

$counterN++; #add another iteration on the cluster counter

}

$cStart = $start1; #set cluster starting position

$cEnd = $end1; #set temporary cluster end postion

$counterNo++; #add another iteration on the cluster counter

@IDs = ''; #flush all values from IDs array, ready for read IDs in the new cluster

}

if ($start1 <= $cEnd+$interval) { #calculate secondary boundary of each cluster

push (@IDs, $ID); #pass reads in clusters to array

#calculate if cluster needs to be expanded or if terminates here

if ($cEnd <= $end1){

$cEnd = $end1;

}

if ($cEnd > $end1){

$cEnd = $cEnd;

}

}

}

#close all files

close (OUT1);

close (OUT2);

close (IN2);

}

print "Script Complete\n"; #terminate script
